# Next-generation smart ophthalmic biomaterials: From passive response to active interaction and closed-loop control

**DOI:** 10.1016/j.bioactmat.2025.10.037

**Published:** 2025-10-30

**Authors:** Pengbo Zhang, Yan Nie, Xiaofang Wang, Xibo Zhang, Longqian Liu

**Affiliations:** a Department of Ophthalmology, West China Hospital, Sichuan University, Chengdu, China; b Laboratory of Optometry and Vision Sciences, West China Hospital, Sichuan University, Chengdu, China; c State Key Laboratory of Southwestern Chinese Medicine Resources, School of Pharmacy, Chengdu University of Traditional Chinese Medicine, Chengdu, China; d Department of Ophthalmology, Affiliated Hospital of Southwest Medical University, Luzhou, 646000, Sichuan, China

## Abstract

Ophthalmic biomaterials are undergoing a rapid transition from inert implants to intelligent, adaptive systems that respond to the spatiotemporal complexity of ocular disease. Conventional materials provide structural support and baseline biocompatibility but rarely adapt to the evolving ocular microenvironment shaped by biochemical and biomechanical cues. Recent advances have delivered stimuli-responsive platforms that release therapeutics or modulate mechanics in response to stimuli such as pH, temperature, enzymatic activity, or mechanical strain. Yet most current strategies remain reactive or preprogrammed, lacking closed-loop, autonomous control. Here we present an evolutionary framework for ophthalmic biomaterials, tracing the shift from passive structures to interactive and emerging closed-loop systems that integrate sensing, on-board decision-making, and actuation. We synthesize advances across the first three generations, delineate core design principles, functional transitions, and clinical implementations, and highlight systems-level integration challenges. Finally, we identify critical opportunities and design principles for intelligent, self-adaptive platforms, providing a conceptual basis for the rational design of next-generation, closed-loop ocular therapies.

## Highlights

• Four-generation roadmap: inert .→ bioactive → responsive → closed-loop.
• Gen IV = sense–compute–actuate platforms for adaptive ocular therapy.
• AI-guided co-design unites materials, sensors, compute, actuators under safety.
• Comparative strengths/limits across generations with clinical status.
• Translation: standard metrics, staged validation, GMP and AI governance.

## Graphical abstract

**Figure:**
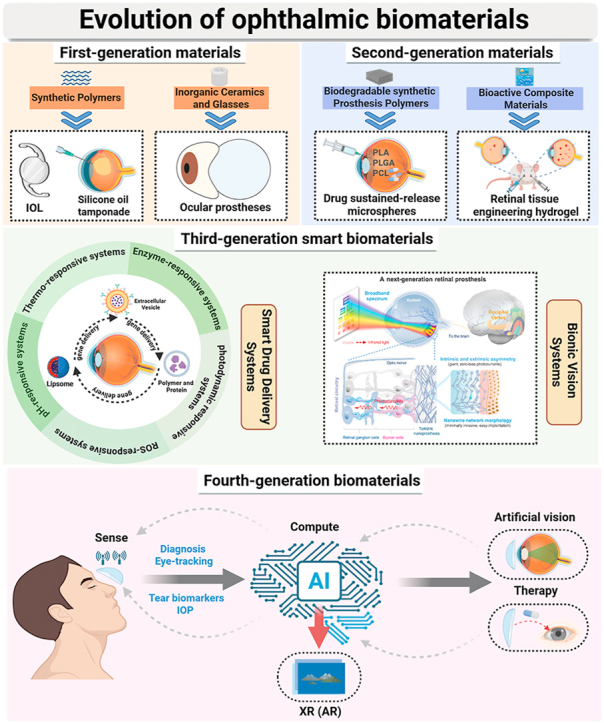

## 1 Introduction

Ocular diseases affect hundreds of millions of individuals globally, often leading to progressive visual impairment and imposing substantial burdens on patient quality of life and health systems alike [1]. Conditions such as glaucoma, diabetic retinopathy (DR), age-related macular degeneration (AMD), cataracts, myopia, and corneal disorders are driven by spatiotemporally evolving pathophysiology that creates dynamically shifting microenvironments [[2], [3], [4]]. These dynamically evolving microenvironments—together with ocular anatomical barriers—make sustained, site-specific therapy difficult [[2], [3], [4], [5], [6]].

Most ocular diseases are largely managed with well-established therapies—pressure-lowering drops and laser or minimally invasive glaucoma surgery for glaucoma [7]; intravitreal anti-vascular endothelial growth factor (VEGF) for neovascular AMD; and, within DR, anti-VEGF with or without corticosteroids for diabetic macular edema, with panretinal or focal laser as an adjunct [[8], [9], [10]]; phacoemulsification with intraocular lenses (IOLs) for cataract [11]; low-dose atropine and optical strategies for myopia [12]; and stepwise medical and surgical management for ocular-surface and corneal disease—lubricants, topical anti-inflammatories/antibiotics, punctal occlusion, amniotic membrane therapies, corneal collagen cross-linking for ectasia, and keratoplasty [[13], [14], [15], [16]]. These approaches are effective for many patients, but their performance is ultimately constrained by short intraocular residence and limited tissue penetration of topical agents, the burden and risks of repeated injections, imperfect spatial selectivity of energy-based or surgical procedures, and variable adherence. As disease microenvironments evolve over time, these constraints create a persistent gap between where drugs are needed and where they actually act.

Biomaterials provide a natural route to close this gap, facilitating controlled drug delivery, supporting tissue regeneration, and enhancing surgical outcomes [[17], [18], [19]]. Early-generation ophthalmic biomaterials primarily served passive structural roles, offering mechanical stability and biocompatibility without interacting with the local microenvironment [20,21]. Within this paradigm, devices such as IOLs, vitreous substitutes, and hydrogel contact lenses (CLs) achieved considerable clinical utility [22,23]. Building on these foundations, second-generation bioactive constructs introduced controlled degradability and interface-level biological activity, For example, collagen-integrated polycaprolactone (PCL) stromal scaffolds that temper fibrosis) [24]. Conventional ophthalmic materials are inherently passive and cannot sense or adapt to evolving biochemical or biomechanical cues, which limits spatiotemporal precision, durability of effect, and safety. These shortcomings underscore the pressing clinical need for smart ophthalmic biomaterials that can actively perceive pathological signals, deliver targeted responses, and autonomously adjust therapeutic actions.

Recent advances in materials science, microfabrication, and bioelectronics are catalyzing a transition toward intelligent ophthalmic systems capable of actively interacting with the ocular milieu. Stimuli-responsive materials that sense environmental triggers—such as pH shifts, enzymatic activity, oxidative stress, temperature, or mechanical strain—now enable spatiotemporally controlled drug release and tunable mechanical behavior [18,25]. In the anterior segment of the eye, emerging minimally invasive platforms—such as responsive microneedle systems and bioactive, self-healing hydrogels—are enabling more localized, durable, and adaptive repair and drug delivery [[26], [27], [28], [29]]. Nonetheless, most third-generation systems remain preprogrammed or stimulus-triggered, lacking the autonomous, real-time control required to adapt therapy to an evolving physiological landscape. The integration of smart materials with microelectronic and wearable platforms supports feedback-controlled therapy, enabling real-time sensing and autonomous adjustment of drug delivery or neuromodulation in response to physiological cues [[30], [31], [32]]. Coupled with embedded computation and artificial intelligence (AI), these systems gain predictive adaptability, dynamically modulating treatment to disease trajectories and optimizing dosing for more personalized ocular care [33]. However, in ophthalmology most implementations remain early prototypes under clinician oversight, and fully verifiable closed-loop control is still in development-these advances indicate a clear path toward precise, durable, and adaptive management, but do not yet achieve it in full.

Here, we introduce an evolutionary framework for smart ophthalmic biomaterials, outlining their progression from passive and reactive systems toward interactive and emerging closed-loop platforms that integrate sensing, on-board computation, and actuation. We delineate core design principles, integration strategies, and clinical frontiers, and highlight how emerging AI technologies could enable precision, autonomy, and personalization in future ocular therapy.

## 2 Evolutionary paradigm of ophthalmic biomaterials

The development of ophthalmic biomaterials has followed a structured evolution-from inert structural replacements to highly adaptive, cell-instructive and biointeractive platforms. This trajectory reflects the convergence of materials science, biomedical engineering, and ocular pathophysiology, with each generational leap addressing specific clinical and technological limitations related to anatomical constraints, dynamic microenvironments, and therapeutic inefficiencies. We delineate a four-stage conceptual model to track the functional evolution and clinical progression of ophthalmic biomaterials (Table 1).

**Table 1:** Translational evolution and clinical progression of ophthalmic biomaterials.

Generation	Representative Materials	Characteristics	Advantages	Limitations	**Ocular Applications**	Clinical Progress/Commercial product
1st: Inert Biomaterials	PMMA, HEMA/PHEMA, PDMS, PPE, PTFE, PVA, YSZ, titanium, gold, platinum	Chemically and mechanically stable; non-degradable; biologically inert	Excellent optical transparency (e.g., PMMA, HEMA/pHEMA), durability, and long-term safety; suitable for structural replacement (e.g., titanium, YSZ)	Lack of bioactivity and tissue integration; low oxygen permeability (e.g., PMMA, hydrogel PVA)	IOLs, KPro, CLs, orbital implants, GDIs, vitreous substitutes	Fully established in clinical practice: PMMA (IOLs: Bausch & Lomb, Bio Vision; KPro: Boston KPro I & II); HEMA/PHEMA (SCLs: Sofclear) PDMS (vitreous substitutes: Silikon 2000), PPE (orbital implants: MEDPOR®, SU-POR®), PVA (eye drops: REFRESH CLASSIC®), PTFE (orbital implants: e.g., ORI II), YSZ (orbital implants: Z7 Zirconia Implant System), titanium (orbital implants: e.g., titanium mesh plates), gold and platinum (e.g., OCULID® Gold Slim/Platinum Slim)
2nd: Bioactive Biomaterials	HAp, β-TCP, Ca-silicate bioceramic, BGs, bioceramic composite, ion-doped bioceramics, SF, Gelatin, Collagen, HA, chitosan, PLGA, PLA, PCL	Biodegradable and surface reactive; controlled dissolution and drug release	Promote cell adhesion, tissue regeneration, and controlled drug delivery; tunable degradation rates	Mechanical weakness; degradation may outpace tissue regeneration; pre-programmed release; no real-time adaptability	Orbital implants and reconstruction, corneal repair, biodegradable GDIs, drug delivery microspheres, anti-scarring membranes, regenerative scaffolds	Clinical stage: Hap (Orbital Implant: e.g., Bio-Eye), BGs (Orbital Implant: S53P4, Porous BG/polyethylene composites), collagen (corneal repair: e.g., SoftShield®, BPCDX), HA (eye drops: e.g., Hylo Gel), PLGA (e.g., Ozurdex®) Pre-clinical stage: β-TCP, Ca-silicate bioceramic, ion-doped bioceramics, SF, Gelatin, chitosan-coated NPs, PLA, PCL
3rd: Actively Interactive Smart Biomaterials	PNIPAM, Pluronic F127, PEG-PLGA, ZIF-8, UCNPs, Ti-Au nanowires, et al.	Stimuli-responsive (pH, ROS, enzyme, light, temperature, mechanics); dynamic release capability	On-demand, spatiotemporal, and targeted drug delivery; improved therapeutic precision	Complex fabrication; mainly open-loop control; potential biosafety concerns	Temperature-sensitive gels, ROS-scavenging nanocarriers, photothermal antibacterial materials , et al.	Clinical stage: Photothermal/photodynamic-responsive biomaterials (intravenous formulation: Visudyne®); artificial retina (e.g., Argus II, Norcom Artificial Retina) Pre-clinical stage: PNIPAM, Pluronic F127, MPC/MMT,PEG-PLGA et al.
4th: Closed-Loop & Autonomous Smart Biomaterials (Emerging)	Zwitterionic hydrogels, conductive nanomaterials (Au nanowires, graphene), biomimetic polymers, iPSC-GelMA hybrids, microdisplay/RFID-integrated interfaces	Integration of sensing, actuation, and computation; mimicry of biological feedback systems	Real-time monitoring and adaptive control; personalized and autonomous therapeutic response	Power and miniaturization limitations; ethical, data, and regulatory barriers	Smart CLs for IOP/tear biomarker/ERG monitoring, on-demand drug delivery, neurostimulation, semi-living corneal scaffolds, autonomous implants	Pre-clinical stage

**First-generation materials** primarily emphasized biocompatibility and mechanical stability, serving as passive structural or optical aids without engaging in biological signaling or tissue interaction. Representative examples include poly(methyl methacrylate) (PMMA) IOLs and early hydrogel CLs. Compared to second-generation materials, these early systems provided reliable structural support but completely lacked bioactive functions for therapeutic intervention.

**Second-generation materials** introduced bioactivity, incorporating drug-eluting capabilities, controlled degradation, and cell-adhesive motifs. These features supported tissue healing and localized pharmacological intervention, marking the transition from inert substitutes to biologically interactive implants. Compared to first-generation passive materials, this represents a fundamental shift toward direct biological engagement. Examples include functionalized hydrogels, anti-scarring membranes, and biodegradable microspheres, marking the initial integration of therapeutic and regenerative functions. However, relative to third-generation systems, their bioactivity is fixed and non-adaptive, and does not respond to dynamic changes in the ocular microenvironment.

**Third-generation smart biomaterials** represented a further evolution toward stimuli-responsiveness, enabling real-time sensing and adaptive drug release in response to local cues such as pH, reactive oxygen species (ROS), enzymes, temperature, or light. Unlike their predecessors, these materials can dynamically respond to environmental changes yet remain reactive rather than autonomously regulated. Compared to second-generation materials, this represents a significant advance in environmental adaptability and spatiotemporal therapeutic precision. Representative systems include pH-sensitive hydrogels for anti-VEGF therapy in DR, thermoresponsive gels for dry eye, and light-activated microneedles for riboflavin delivery in corneal crosslinking—achieving spatiotemporally precise, minimally invasive intervention but externally dependent interventions. However, compared to fourth-generation systems, they lack the integrated sensing-processing-actuation loops required for autonomous regulation and closed-loop therapeutic control.

**Fourth-generation biomaterials**, now in early translation, move beyond environmental responsiveness toward closed-loop therapeutic control by integrating multimodal sensing, on-board processing, and autonomous actuation. Compared to third-generation stimulus-responsive materials, this represents a paradigm shift from reactive adaptation to proactive, self-regulated therapeutic management. Smart CLs exemplify this trend by integrating multimodal sensing-continuous introcular pressure (IOP) monitoring, tear-biomarker analytics, electroretinography (ERG) ocular-biopotential readout, and radio frequency (RF)-tag-based eye tracking with a split-compute architecture supported by wireless power and telemetry, enabling on-demand actuation such as triggered drug release or optical/electrical stimulation. Although not yet fully autonomous or AI-driven, such integration supports feedback-mediated actuation and advances toward homeostasis-mimicking ocular platforms across wearable and implantable modalities.

## 3 First-generation inert ophthalmic biomaterials

First-generation biomaterials are defined as inert, biocompatible materials primarily designed to replace or support damaged ocular structures without interacting biologically with surrounding tissues. These early ophthalmic materials were engineered to minimize chemical and biological interactions with ocular tissues, prioritizing mechanical stability and biocompatibility for long-term use. Compared with contemporary bioactive systems, these materials exhibit limited biological integration. Nevertheless, owing to their chemical stability, mechanical strength, and established safety profiles, they remain clinically relevant across multiple ophthalmic indications.

### 3.1 Synthetic polymers

#### 3.1.1 Poly(methyl methacrylate) (PMMA)

**Fig. 1:**
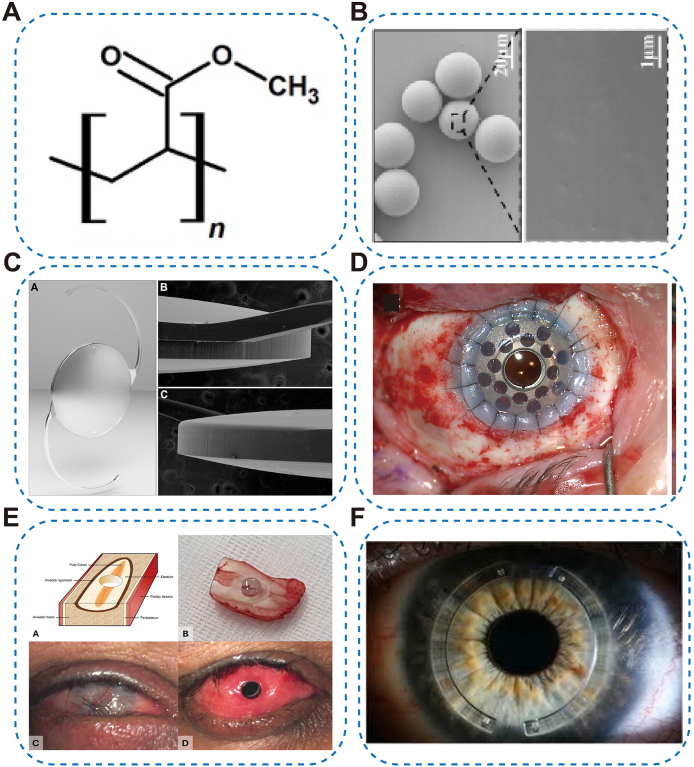
Representative ophthalmic applications of PMMA. (A) Chemical structures of PMMA [44], copyright 2019, Materials (Basel); (B) Characterization of PMMA Microspheres [235], copyright 2025, Bioactive Materials; (C) Single-piece, square-edge PMMA IOL featuring a 0.4-mm optic vault, a continuous 360° posterior square edge, a rounded anterior edge, and a truncated posterior edge [38], copyright 2017 Ophthalmology; (D) Boston Type I KPro implanted after 270° mucosal flap resection, with the inferior mucosa retained as a hinge [39], copyright 2025, American Journal of Ophthalmology; (E) Osteo-odonto-kPro implant [40],copyright 2025, American Journal of Ophthalmology; (F) Example of PMMA-intrastromal corneal rings implanted in the eye [43], copyright 2019, Cochrane Database of Systematic Reviews.

PMMA is an amorphous, glassy, hydrophobic poly(methacrylate). Its dense, non-porous network and high Tg confer polishable optical clarity, dimensional/chemical stability, and long-term biostability; conversely, very low oxygen transmissibility (Dk/t) and poor wettability limit corneal wear, and the rigid matrix precludes foldability [34]. Clinically, PMMA has served as a foundational material for several ophthalmic devices (Fig. 1A and B) [35,36]: it was a principal IOLs optic but has been largely superseded by foldable acrylics because it requires large incisions (Fig. 1C) [37,38]; it remains the transparent core in keratoprostheses (KPro), such as Boston KPro and OOKP, where clarity and inertness are critical (Fig. 1D and E) [[39], [40], [41]] and it is used in intrastromal corneal ring segments for keratoconus (Fig. 1F) [42,43]. Historically, rigid PMMA contact lenses corrected refractive error and ectasia but yielded to higher-oxygen permeability (Dk) silicone-acrylate rigid gas-permeable (RGP) lenses due to PMMA's low Dk [44]. Strengths: excellent machinability, optical quality, and durability. Limitations: non-foldability, low Dk and wettability (risking hypoxia with corneal wear), and inferior capsular behavior versus modern hydrophobic acrylic IOLs.

#### 3.1.2 Acrylic Polymers

**Fig. 2:**
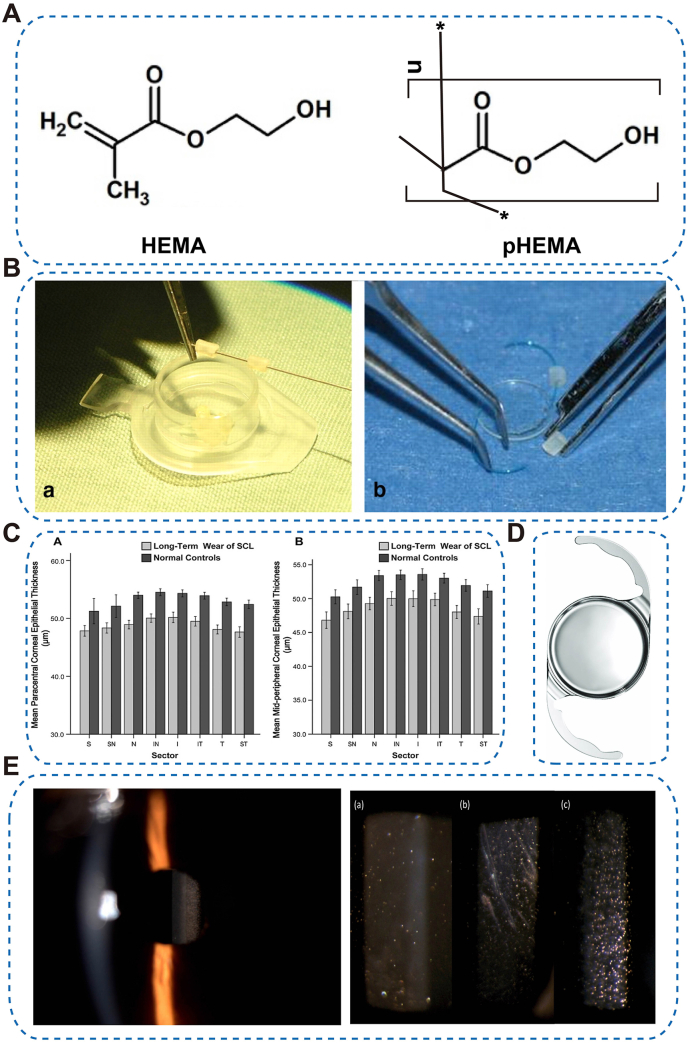
Representative ophthalmic applications of Acrylic Polymers. (A) Chemical structures of HEMA and pHEMA [44,236], copyright 2019, Materials (Basel), copyright 2015, International Journal of Pharmaceutics; (B) A molded pHEMA hydrogel contact lens placed intraoperatively onto IOL haptics [46], copyright 2011, Investigative Ophthalmology and Visual Science; (C) Comparison of epithelial thickness profiles in the paracentral and midperipheral cornea, showing sectoral differences between soft contact lens wearers and non-wearers [49], copyright 2014, Optometry and Vision Science; (D) A new hydrophobic acrylic IOL: CT LUCIA 611P [51], copyright 2022, Journal of Cataract and Refractive Surgery; (E) Examples of glistenings in AcrySof (Alcon) IOLs at varying severities and implantation durations, including a yellow-tinted model at 6 months post-implantation and severe cases several years after implantation, copyright 2014, Clinical Ophthalmology, copyright 2015, Current Eye Research.

Hydrophilic acrylics (HEMA/pHEMA) are water-absorbing transparent polymers. Hydrophilic methacrylate networks (e.g., HEMA → pHEMA) form cross-linked, water-rich matrices with tunable mesh size, which increase surface wettability and improve oxygen transport relative to rigid, hydrophobic PMMA (Fig. 2A) [44,45]. pHEMA is widely used in soft CLs (SCLs) and has been explored in foldable IOLs (Fig. 2B) [35,46]. Copolymerization fine-tunes performance: methacrylic acid (MAA) introduces carboxyl groups that raise water content and ionic character (enhancing wettability but with greater dehydration/protein-deposition susceptibility), whereas N-vinylpyrrolidone (NVP) increases hydrophilicity and optical clarity with less ionic load; moderate ethylene glycol dimethacrylate (EGDMA) cross-linking boosts modulus and shape retention by tightening mesh size, while excessive cross-linking reduces swelling/oxygen permeability and increases brittleness [44,47,48]. Advantages: softness/conformability for minimally invasive implantation and comfort. Limitations: restricted tear exchange and dehydration of high-water gels can promote corneal adhesion and epithelial compromise during prolonged wear (Fig. 2C) [49,50].

Hydrophobic acrylics are low-water, high-refractive-index polymers. Low-water (<1 %) methacrylate polymers with high refractive index (≈1.44–1.55)—including poly(2-phenylethyl methacrylate) (polyPEMA), poly(ethyl methacrylate) (polyEMA), and poly(2,2,2-trifluoroethyl methacrylate) (polyTFEMA)—provide dimensional stability and biodegradation resistance, and now dominate modern foldable IOLs (Fig. 2D) [45,51,52]. Advantages: excellent capsular biocompatibility and lower posterior capsule opacification (PCO) versus hydrophilic acrylics and PMMA [53]. Limitations: formation of “glistenings” (fluid-filled microvacuoles) can degrade optical quality at high densities (Fig. 2E) [54,55].

#### 3.1.3 Polydimethylsiloxane (PDMS)-Based materials

**Fig. 3:**
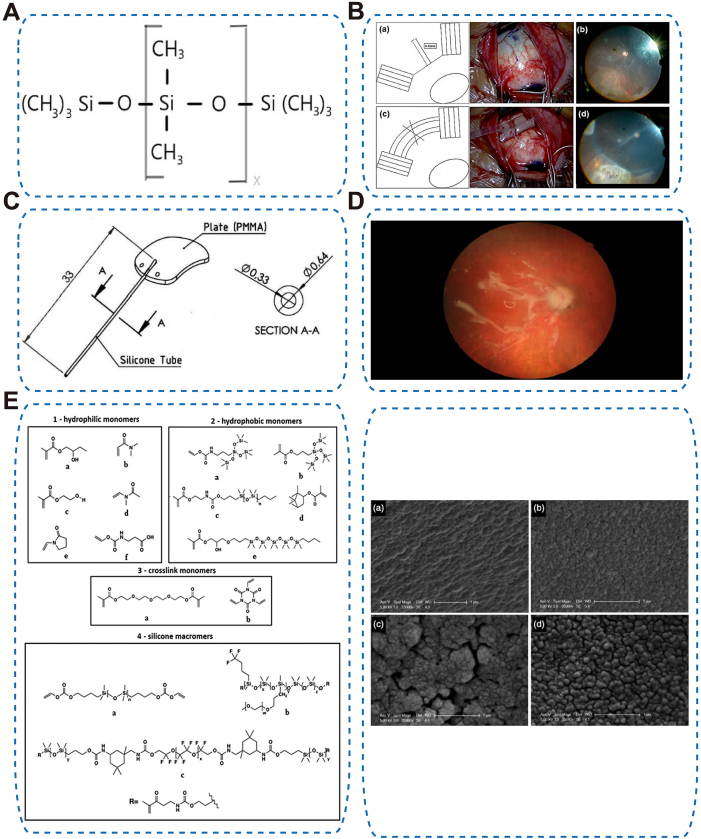
Representative ophthalmic applications of PDMS-Based Materials. (A) Chemical structures of PDMS [237], copyright 2024, Materials (Basel); (B) Scleral buckling procedure using silicone tire (#287) and circumferential band (#240) with sleeve (#270), showing pre-and postoperative fundus images of rhegmatogenous RD [238], copyright 2025, PLoS One; (C) Schematic representation of a valved glaucoma implant (VGI), copyright 2025, Clinical and Experimental Ophthalmology; (D) Optic disc swelling observed with HeavySil tamponade [62], copyright 2014, Biomed Research International; (E) Chemical structures of monomers used in four commercial silicone hydrogel lenses and ESEM images of hydrated lenses: (a) narafilcon A, (b) lotrafilcon B, (c) comfilcon A, (d) balafilcon A [239], copyright 2021, Journal of Biomedical Materials Research-Part B: Applied Biomaterials.

PDMS is a versatile silicon-based polymer used as elastomers, fluids (silicone oils, SOs), and—via composite approaches—hydrogels (Fig. 3A).

**Silicone elastomers** are flexible, biocompatible polymers composed of crosslinked polysiloxane chains, widely used in scleral buckles and glaucoma drainage devices (GDIs) [22,56] (Fig. 3B–C). Their low-modulus, smooth, chemically stable surface conforms to ocular tissues with minimal protein/cell adhesion. In rhegmatogenous retinal detachment (RD), silicone buckles relieve vitreoretinal traction and promote reattachment [57]. Similar advantages allow its use in GDIs [56]. Its low inflammatory potential, superior tissue integration, and smooth surface-exemplified by Ahmed valves-reduce leukocyte and collagen adhesion, helping to limit fibrosis [56]. Silicone elastomers were also used in IOL optics [52], but are now less common due to higher PCO rates and adhesion to intraocular SOs [22,58].

**SOs** are high-molecular-weight PDMS fluids used as intraocular tamponades in complex retinal detachments [59]. Structurally, SOs consist of linear siloxane chains with tunable viscosity (commonly 1000–5000 mPa s) and, in **heavy SOs (HSOs)** (Fig. 3D), semi-fluorinated alkane blends to increase density. Functionally, they provide tamponade support to maintain retinal attachment. Advantages include biocompatibility and customizable physical properties for tamponade efficacy; limitations involve handling challenges at higher viscosities and increased emulsification risk in denser or low-viscosity formulations [[60], [61], [62], [63]]. Modified formulations, such as Siluron Xtra® and Siluron 2000, optimize the balance between stability, surgical manageability, and tamponade performance [64].

**Silicone hydrogels** are composite materials combining oxygen-permeable silicone networks with hydrophilic hydrogel components. Structurally, they integrate silicone for high oxygen transmissibility with hydrogel chains to enhance wettability. Functionally, these materials provide mechanical robustness and comfort for daily and extended-wear SCLs [44] (Fig. 3E). Although their intrinsic hydrophobicity can compromise wettability and comfort, surface treatments and internal wetting agents mitigate these effects [65].

#### 3.1.4 Polytetrafluoroethylene (PTFE)

PTFE, a highly fluorinated and highly crystalline polymer, is exceptionally chemically inert with very low surface energy. As a nonporous film it integrates poorly, whereas expanded PTFE (ePTFE) with a node–fibril architecture and interconnected pores ≥∼20 μm permits keratocyte infiltration and ECM deposition, producing durable stromal interlock. ePTFE has been used in custom keratoprosthesis components as a porous skirt/backplate because of its chemical inertness and microstructure that supports stromal integration [22,66,67].

#### 3.1.5 Porous polyethylene (PPE)

Porous polyethylene (PPE) is a sintered form of high-density polyethylene engineered with interconnected pores to enable fibrovascular ingrowth and tissue integration.

PPE, e.g., Medpor® is a widely used orbital implant material for volume restoration after enucleation, fracture repair, or congenital deformities [68]. Its porosity allows fibrovascular ingrowth to stabilize the implant. Compared with hydroxyapatite (HAp), PPE offers greater mechanical strength, easier handling, lower cost, and reduced inflammatory response, though vascularization is slower [69]. Complications, occurring in ≈10 % of cases, include exposure, infection, and chronic inflammation [70], which may be mitigated by techniques such as autologous wrapping.

#### 3.1.6 Polyvinyl alcohol (PVA)

PVA is a water-soluble synthetic polymer. Hydroxyl-rich, semicrystalline PVA forms hydrogen-bonded, water-rich networks that are lubricious and resist protein adsorption; crystalline domains impart good tensile strength, but the lack of gas-permeable silicone segments yields low oxygen permeability. PVA is primarily used in SCLs due to its biocompatibility, high hydrophilicity, and low protein adsorption [71]. Studies have shown that PVA hydrogels process greater tensile strength and reduced protein absorption compared to other CLs materials, such as silicone. However, similar to PMMA, limited oxygen permeability restricts its performance in extended-wear applications unless modified [72].

### 3.2 Inorganic ceramics and glasses

Bioceramics are dense inorganic materials with high hardness, strength, and wear resistance. Surface properties and porosity govern cell and tissue integration. Examples include alumina [[73], [74], [75]], and yttria-stabilized zirconia (YSZ), widely used in orbital implants for their biocompatibility and low bacterial adhesion, though brittleness can limit load-bearing use.

Traditional glass, with its amorphous Si–O network, offers excellent transparency and chemical stability but low fracture toughness and limited surface tunability. Once widely used in ophthalmic devices [76], including KPro and ocular prostheses [77,78], it has largely been replaced by advanced polymers and composites.

### 3.3 Metals and metallic alloys

Metallic implants are dense, ductile materials commonly used in periocular reconstruction, particularly for exposure keratopathy and facial nerve palsy. Their structure, based on metallic bonding and surface passivation, provides high strength, corrosion resistance, and radiopacity. Functionally, gold and platinum are employed for upper-eyelid loading to restore passive closure by increasing gravitational force [79], while titanium supports lower-eyelid structures and orbital frameworks due to its high strength-to-weight ratio and biocompatibility [80]. Advantages of these materials include mechanical durability, precise load-bearing, biocompatibility, and, for platinum, MRI compatibility and intraoperative malleability. Limitations involve potential fibrotic encapsulation, implant exposure, and, in the case of gold, bulkiness that may cause contour abnormalities or imaging artifacts, necessitating careful surgical planning and, in some cases, surface modification to optimize tissue integration.

## 4 The second-generation ophthalmic bioactive materials

Second-generation biomaterials are defined as biofunctional materials designed to achieve controlled degradability and active biological modulation. This represents a fundamental shift from the passive biocompatibility of first-generation materials toward direct biological engagement. The central advance was achieving high tunability at the material–tissue interface to actively guide repair and regeneration [81,82]. Representative systems include bioactive ceramics, biodegradable polymers, and composites [82] (Table 2). Their adoption shifted ophthalmic interventions from purely structural support toward biologically integrated therapies and laid essential groundwork for third-generation intelligent biomaterials.

**Table 2:** Representative 2nd bioactive biomaterials in ophthalmology: Functions and applications.

Material Class	Examples	Key Functions	Ocular Applications	Advantages	Limitations
Bioactive ceramics	HA, β-TCP, BGs	Osteoconduction, angiogenesis	Orbital implants	Rapid vascularization, low immune response	Brittle, poor infection resistance
Element-doped ceramics	Cu-BG, Zn-wollastonite	Antibacterial, pro-angiogenic	Orbital scaffolds, KPro	Enhanced bioactivity	Doping precision and safety
Natural polymers	SF, gelatin, collagen, HA, chitosan	ECM mimicry, cell adhesion	Corneal scaffolds, drug carriers	Biocompatible, regenerative	Poor mechanics, complex fabrication
Synthetic polymers	PLGA, PLA, PCL	Controlled degradation, drug delivery	Sustained-release microspheres/films/implants; nanofiber scaffolds; drug delivery	Tunable kinetics, scalable	Burst release; Brittleness (PLA) or excessive hydrophobicity (PCL)
Composite systems	BG-polymer hybrids, gelatin-PCL	Multifunctional, mechanical integrity	Anti-infective membranes, stromal repair	Dual function: support + bioactivity	Still lack dynamic responsiveness

### 4.1 Bioactive ceramics

As a representative class of second-generation materials, bioactive ceramics mainly include calcium phosphate (CaP) materials (e.g., HAp, tricalcium phosphate-TCP), calcium silicate ceramics, and bioactive glasses (BGs) [83]. With excellent biocompatibility, intrinsic bioactivity, and osteoconductivity, these materials are widely used in orbital implants, orbital reconstruction, and KPro [[84], [85], [86], [87], [88]].

#### 4.1.1 Hydroxyapatite (HAp)

**Fig. 4:**
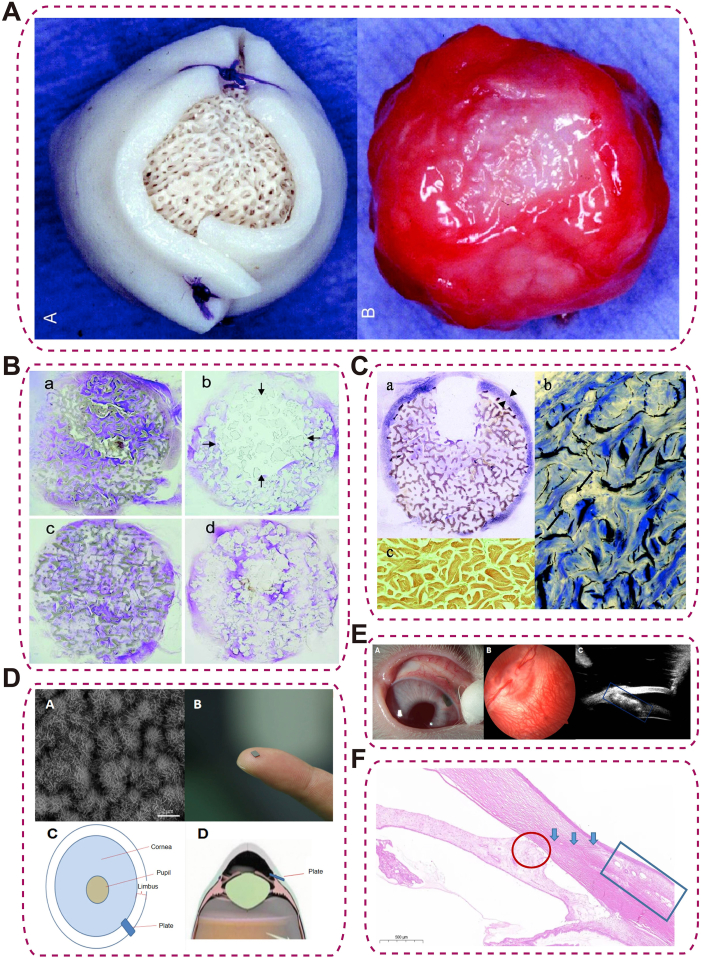
HAp-based ocular implants and tissue integration. (A) HAp implant wrapped with acellular dermis before surgery (left) and PPE implant harvested 12 weeks after surgery showing intact dermis wrap (right); (B) Histologic sections of HAp and PPE implants at 6 weeks (a, b) and 12 weeks (c, d) demonstrating progressive vascularization; incomplete vascularization in PPE at 6 weeks indicated by arrows; (C) Low- and high-magnification views of HAp implant showing intact dermis wrap (arrowheads) and vascular ingrowth (arrows); control dermis shows no vascularization or inflammation [90], copyright 2024, Ophthalmic Plastic and Reconstructive Surgery (A–C); (D) SEM image of HAp coating surface and schematic of glaucoma drainage plate placement; (E) Anterior and posterior segment examinations and fixation of the drainage plate; (F) Histology of HAp–Mg glaucoma drainage plate showing aqueous humor drainage channel (blue arrows), anterior synechia (red circle), and microcystic filtering bleb (blue rectangle) [92], copyright 2024, ournal of Materials Science: Materials in Medicine (D–F).

HAp is a naturally occurring calcium phosphate mineral and the main inorganic component of bone, characterized by excellent biocompatibility, osteoconductivity, and chemical similarity to bone, enabling rapid fibrovascularization, stable anchorage, and durable host–implant integration for orbital implants [89]. In vivo, HAp embedded within acellular dermal matrices has been reported to achieve rapid fibrovascularization (≈6 weeks), faster than PPE, with minimal inflammation (Fig. 4A–C), while maintaining structural stability for at least 12 weeks to promote durable host–implant integration [90,91]. This vascularization underpins stable anchorage, enhanced prosthesis motility, and reduced risks of migration or socket contracture. Extending beyond structure, HAp's modifiability enables next-generation devices; for example, HAp-coated magnesium (HAp-Mg) GDIs degraded safely over ≈4 months in animal models, lowering IOP without corneal damage or overt inflammation [92] (Fig. 4D–F). However, limitations involve brittleness and low mechanical strength, which can restrict load-bearing applications.

#### 4.1.2 Tricalcium phosphate (TCP)

**Fig. 5:**
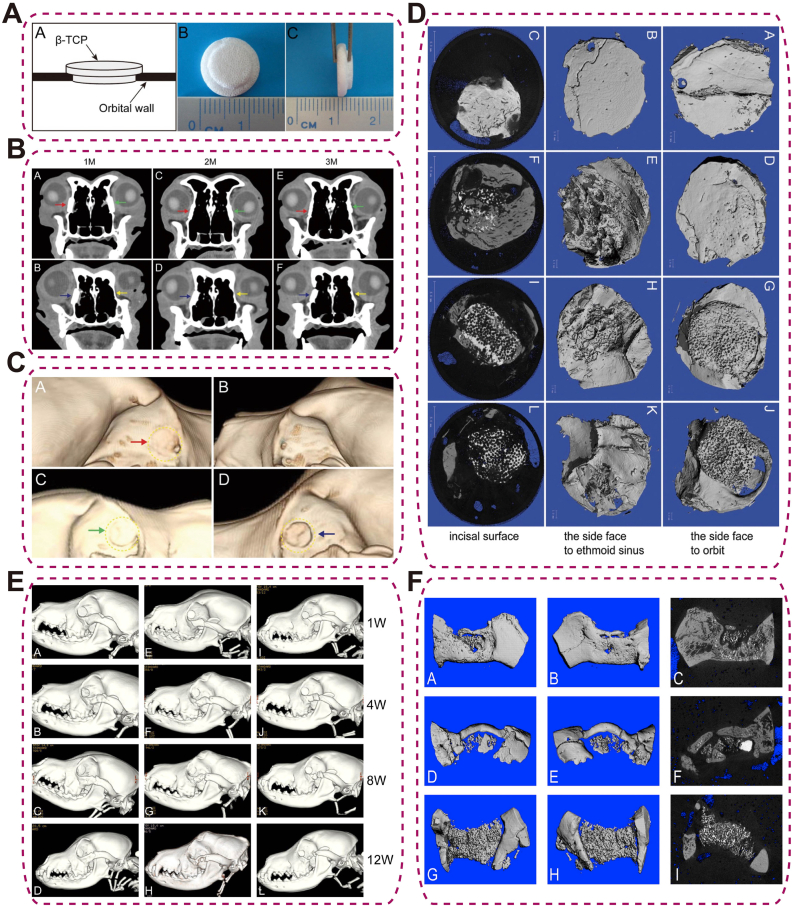
Repair of orbital wall defects using BMSCs/β-TCP in canine models. (A) “Plug”-shaped β-TCP frame for orbital wall repair; (B) CT coronal views at 1, 2, and 3 months, showing induced BMSC/TCP (red), noninduced BMSC/TCP (green), TCP (blue), and normal (yellow) groups; (C) 3D-CT and (D) micro-CT at 3 months post-repair [94], copyright 2013, Journal of Biomedical Materials Research - Part B: Applied Biomaterials (A–D); (E) 3D-CT at 1, 4, 8, and 12 weeks, and (F) micro-CT at 12 weeks postoperatively [95], copyright 2011, Investigative Ophthalmology and Visual Science (E–F).

TCP is a biodegradable calcium phosphate ceramic available as α- and β-polymorphs, with a lower Ca/P ratio than HAp, leading to faster resorption in vivo [93]. Rely on its high biodegradability and osteoconductivity, in orbital bone repair, β-TCP scaffolds seeded with osteogenically induced autologous bone marrow stromal cells (BMSCs) supported robust regeneration in canine models. For 10-mm medial wall defects, only the induced BMSC/β-TCP group achieved effective repair, with new bone bridging the defect toward the ethmoidal sinus and complete mucosal coverage (Fig. 5A–D) [94]. Similarly, 25-mm inferior rim defects repaired using 3D β-TCP scaffolds with induced BMSCs formed extensive trabeculae, bony union, and near-native density by 12 weeks, whereas non-induced or scaffold-only groups showed incomplete healing (Fig. 5E and F) [95]. Despite its osteogenic potential, TCP's rapid resorption rate and inherent mechanical weakness limit its use in critical-sized, load-bearing defects.

#### 4.1.3 Ca-silicate bioceramic

**Fig. 6:**
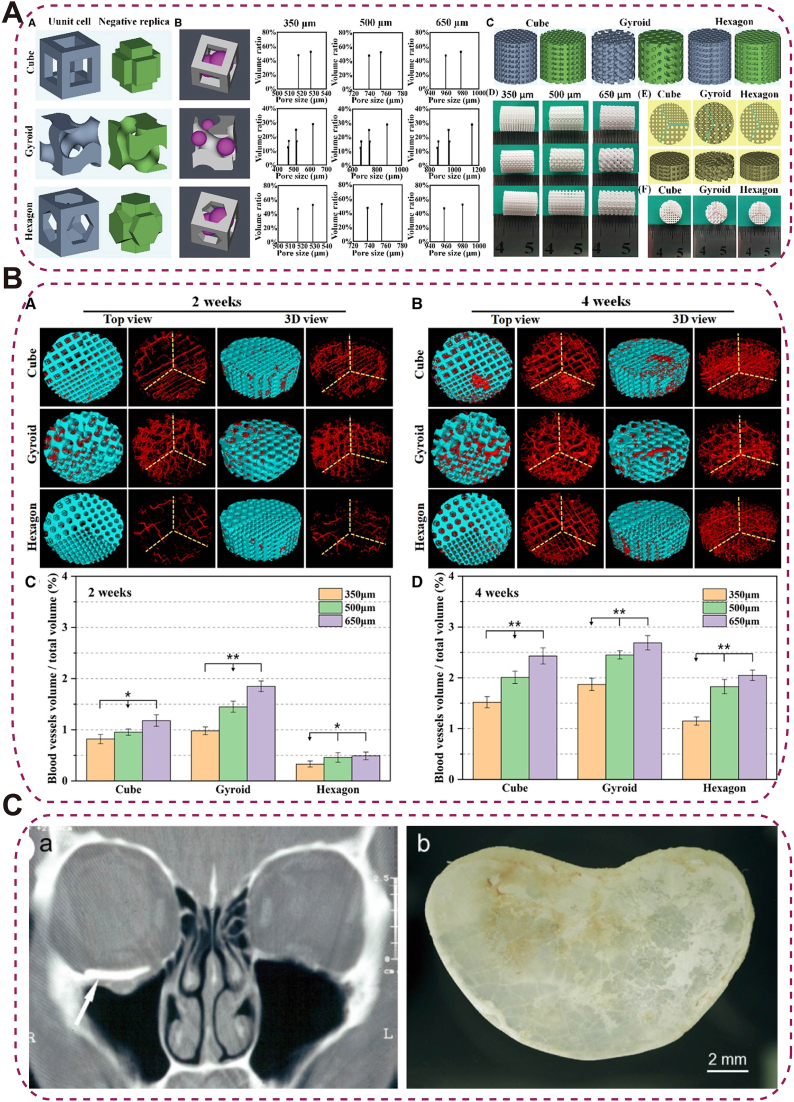
Ca-Silicate and BGs Scaffolds for Orbital Bone Regeneration. (A) 3D-printed Ca-silicate bioceramic scaffolds with cube, gyroid, and hexagon pore geometries, illustrating pore size definition, negative replicas, and as-sintered appearances at various dimensions; (B) Micro-CT 3D reconstructions of vascularization within Ca-silicate scaffolds at 2 and 4 weeks post-implantation, with quantitative BV/TV analysi [96], copyright 2021, Regenerative Biomaterials (A–B); (C) Bioactive glass (BG) plate for orbital floor repair: CT scan and retrieved plate 2 years post-surgery [100], copyright 2008, Journal of Oral and Maxillofacial Surgery.

Ca-silicate bioceramics are bioactive calcium-silicate–based materials that support bone and tissue integration. Si-rich phases (e.g., wollastonite, Ca-silicates) release SiO_4_^4−^/Ca^2+^ to enhance alkaline phosphatase (ALP) activity, collagen/mineral deposition, and enable faster, tunable degradation than HAp. Mg-doped variants (e.g., CSI-Mg8) combine bioactivity, controlled degradation, and precision 3D-printed porosity for improved performance. Gyroid architectures promote early vascular ingrowth through curved surfaces and favorable ion release profiles, albeit at some expense to mechanical strength (Fig. 6A and B). These features position Mg-doped calcium silicates as promising orbital implants that balance biological performance with structural demands [96]. Nevertheless, the enhanced bioactivity achieved through engineered porosity inevitably compromises mechanical strength.

#### 4.1.4 Bioactive glasses (BGs)

BGs—including S53P4 silicate glass, borosilicates, and copper-doped mesoporous coatings—are silicate- or borosilicate-based materials that release bioactive ions (e.g., Ca^2+^, PO_4_^3−^, Si^4+^) that stimulate osteogenesis and form an HAp-like layer for bonding to host tissue (Fig. 6C) [97,98]. They offer strong bioactivity, bone integration, and long-term stability but are limited by brittleness, shaping difficulty, and pH fluctuations. In orbital floor fractures, melt-cast S53P4 plates have shown favorable outcomes with robust bone formation and long-term implant integration [99]. Clinical series from Finland reported lower complication rates and superior functional/aesthetic results versus autologous cartilage; CT follow-up revealed persistent new bone tightly apposed to the implant beyond two years [100].

#### 4.1.5 Element-doped and composite bio-ceramics

Functional modifications to traditional bioceramics—including elemental doping and composite architectures—have significantly expanded their applicability in ophthalmology. These approaches aim to integrate mechanical, biological, and pharmacological functions into a unified scaffold.

Bioceramic composites—such as glass-reinforced HAp, BG/polymer hybrids, and BG-ceramic–coated metals—enhance strength and biocompatibility in ocular implants. To address KPro complications (epithelial ingrowth, membrane formation, secondary glaucoma), titanium-based prostheses coated with BG-ceramic composites have reduced epithelial invasion into the anterior chamber and helped prevent infection/dislocation in rabbit models [101]. Porous BG/polyethylene composites have shown clinical safety in KPro recipients, though limited angiogenesis and infection resistance remain concerns [102]. However, bioceramic composites can still suffer from brittleness, interfacial mismatch, and complex fabrication processes that limit their clinical translation.

**Fig. 7:**
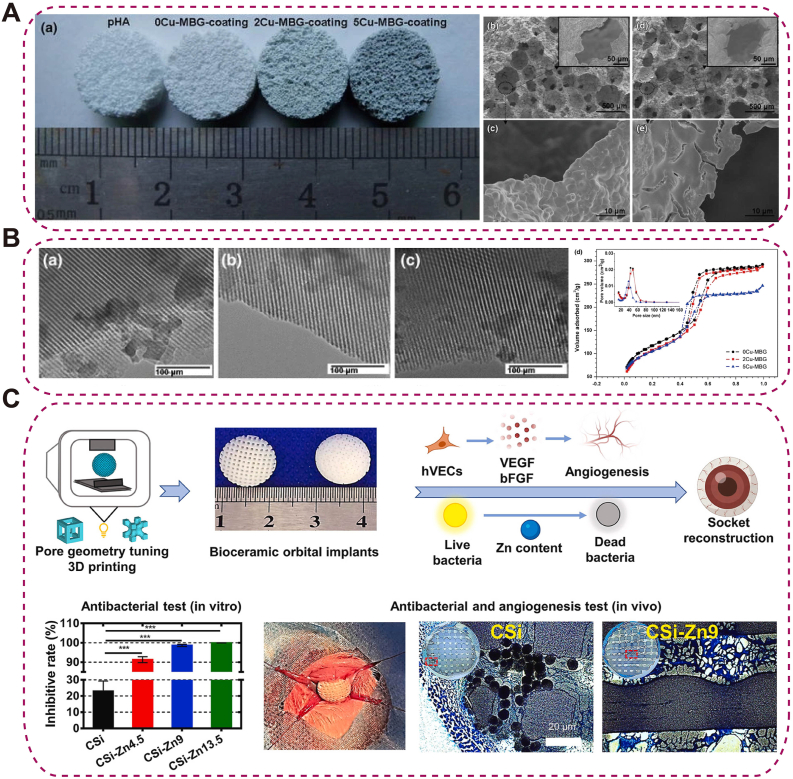
Element-doped and composite bio-ceramics for orbital applications. (A) Porous HAp scaffolds before and after xCu-MBG coating, showing crystalline HAp grains and Cu-MBG layers on pore walls; (B) TEM of 0Cu-, 2Cu-, and 5Cu-MBG coatings with N_2_ adsorption-desorption and Barrett-Joyner-Halenda (BJH) pore-size distribution [103], copyright 2014, Biotechnology Letters (A–B); (C) Zn-doped wollastonite bioceramics exhibiting enhanced angiogenic and antibacterial properties for orbital reconstruction [104], copyright 2024, Bioactive Materials.

To overcome these issues, ion-doped bioceramics are attracting attention. CuO-containing mesoporous BG (Cu-MBG), engineered with nanoscale channels for sustained Cu^2+^ release, improved vascular infiltration and provided intrinsic antibacterial activity [103] (Fig. 7A–B). Zn-doped wollastonite fabricated by stereolithography exhibited >90 % antibacterial efficacy and promoted fibrovascular integration in rabbit orbital-implant models [104] (Fig. 7C). Collectively, their tunable composition supports better integration with surrounding tissues, making them valuable for ocular and orbital reconstruction. However, excessive ion release may induce cytotoxicity or local pH imbalance, and maintaining uniform ion distribution and long-term stability remains challenging.

### 4.2 Biodegradable polymers

**Fig. 8:**
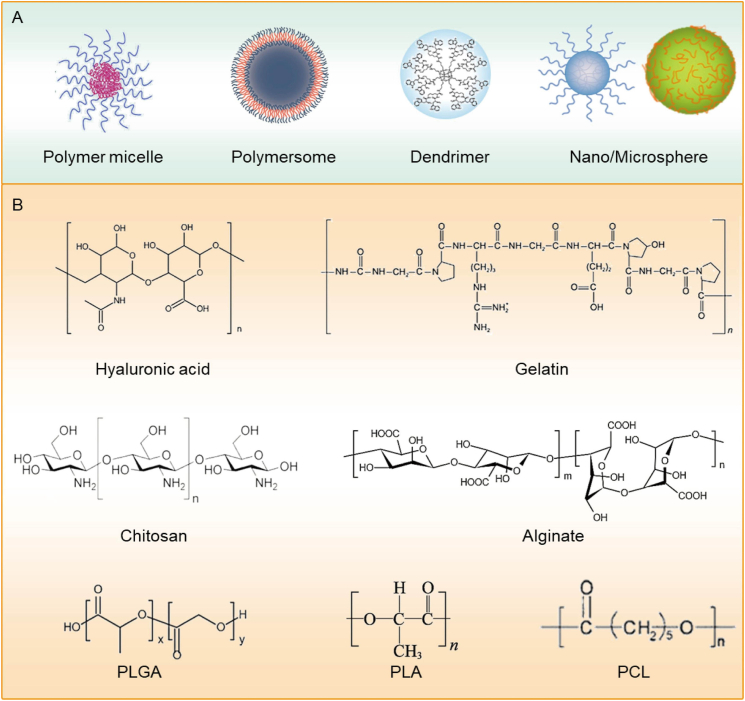
Structural diagrams of polymeric materials for ocular disease treatment. (A) Simplified schematic of polymeric particles for ocular drug delivery; (B) Representative chemical structures of compounds commonly used to prepare polymeric particles [25], copyright 2023, Advanced Drug Delivery Reviews (A–B).

Biodegradable polymers used in ophthalmology can be broadly categorized into natural and synthetic types. Natural polymers—such as chitosan, gelatin, collagen, and hyaluronic acid (HA)—are valued for their excellent biocompatibility, inherent bioactivity, and their ability to mimic the extracellular matrix (ECM), making them especially suitable for promoting cell adhesion and tissue regeneration [105,106]. In contrast, synthetic polymers like polylactic acid (PLA), poly(lactic-co-glycolic acid) (PLGA), and PCL offer greater control over mechanical strength, degradation rates, and structural design. Both types of materials are widely used in ocular drug delivery and regenerative medicine due to their tunable biodegradability and ease of functional modification (Fig. 8A and B) [107,108].

#### 4.2.1 Natural polymers

**Fig. 9:**
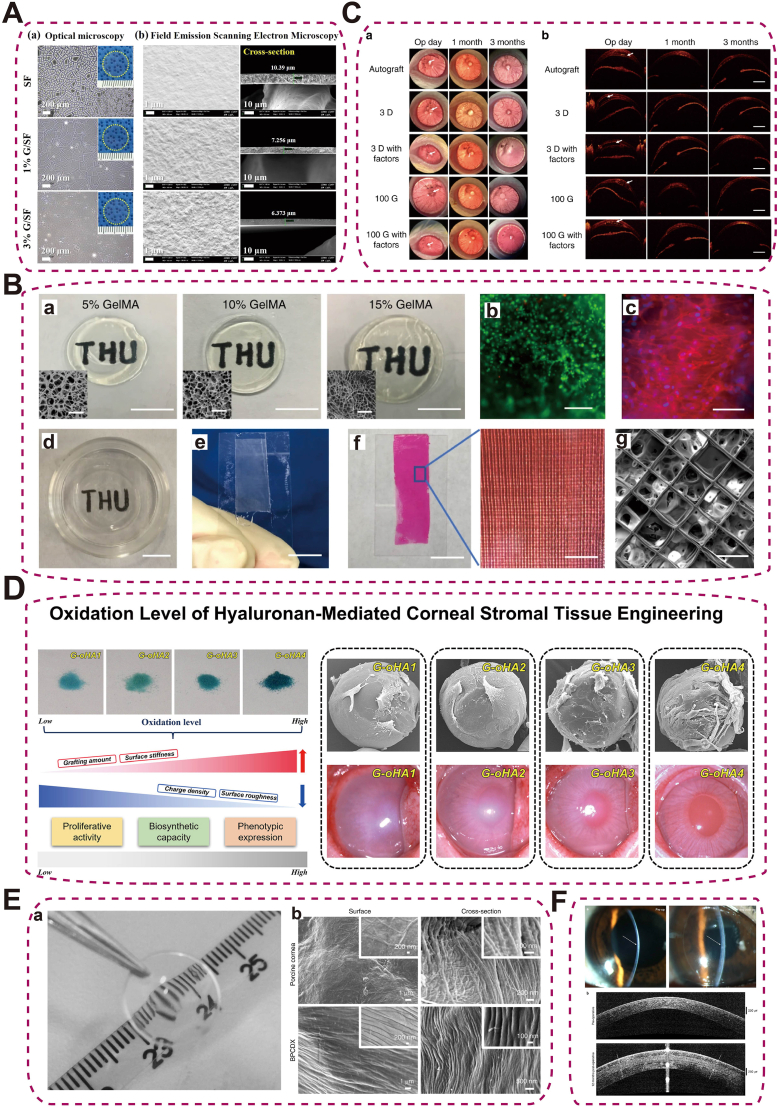
Natural polymer-based biomaterials for corneal tissue engineering.(A) Optical and FESEM images of SF films with 0 %, 1 %, and 3 % glycerol-SF (GSF), showing surface morphology and cross-sectional thickness [111], copyright 2019, Journal of Biomaterials Science, Polymer Edition; (B) GelMA hydrogels with varying concentrations (5 %, 10 %, 15 %) showing transparency and microstructure, cell viability, cytoskeleton staining, and 3D bioprinted fiber-reinforced constructs; (C) In vivo engraftment of fiber-reinforced GelMA hydrogel promoting stromal matrix regeneration in rat cornea [120], copyright 2020, Nature Communications (B–C); (D) Oxidation Level of Hyaluronan-Mediated Corneal Stromal Tissue Engineering [106], copyright 2022, Carbohydrate Polymers; (E) Transparent, curved BPCDX with SEM images showing tightly packed collagen fibrils slightly thicker than those in native porcine cornea; (F) Slit-lamp images before and 1 day after implantation, highlighting immediate increases in central corneal thickness and curvature [124], copyright 2023, Nature Biotechnology (E–F).

Silk fibroin (SF) is a natural protein polymer extracted from silk that comprises β-sheet crystallites embedded in amorphous chains; higher β-sheet content confers high tensile strength and protease resistance. SF has been processed into hydrogels, membranes, and porous scaffolds for ocular surface repair [109,110]. Glycerol/SF (G/SF) membranes improve flexibility and biocompatibility, supporting corneal endothelial adhesion and proliferation (Fig. 9A) [111]. Additional variants—tropoelastin‐reinforced SF, gelatin-blended SF with accelerated degradation (≈90 % mass loss within 48 h), and electrospun PCL–SF nanofibers to enhance transparency, hydrophilicity, and strength—have been explored [[112], [113], [114]]. Limitations include a slight yellow tint, slow degradation, brittleness, and processing complexity (electrospinning/chemical crosslinking/methacrylation + UV). Future work should streamline fabrication, accelerate controlled degradation, and augment bioactivity.

Gelatin, a collagen-derived protein, is ECM-mimetic but has low mechanical strength and rapid degradation, motivating chemical modification or composites [115,116]. o-Nitrobenzaldehyde–modified gelatin (GelNB) affords light-activated gels that promote corneal cell migration and wound healing [117]. Methacrylated gelatin (GelMA) tunes mesenchymal stromal cells (MSCs) behavior by concentration (7 wt% promotes proliferation; 30 wt% biases corneal differentiation) [118], and 12.5 wt% GelMA enables high-resolution 3D printing of stromal constructs with excellent compatibility [119]. Composites that integrate GelMA with PCL–polyethylene glycol (PEG) microfibrous scaffolds recapitulate stromal lamellae, improve mechanics, and maintain keratocyte phenotype and stromal regeneration (Fig. 9B and C) [120]. In vivo, oxidized HA–grafted gelatin microcarriers reduced edema, restored collagen, and repaired stroma in alkali-burned rabbits (Fig. 9D) [106]; ascorbic-acid–loaded GelMA enhanced collagen synthesis and stromal repair [121,122]. Gelatin/PCL composite membranes combine mechanical reinforcement with antibacterial performance via sustained gentamicin release [123], highlighting the potential of multifunctional composites in ophthalmic therapy. Practical constraints include fabrication-induced protein denaturation, batch variability, and overly fast degradation.

Collagen provides an ECM-mimetic milieu that supports epithelial regeneration and facilitates solute diffusion. A double-crosslinked porcine collagen construct (BPCDX) forms an implantable transparent hydrogel; in a clinical study of advanced keratoconus (n = 20), BPCDX increased corneal thickness by 209–285 μm and maintained transparency and enzymatic resistance over 24 months (Fig. 9E and F) [124]. Collagen membranes embedded with AuNP–miR-133b conjugates (physically crosslinked) reduced stromal scarring in a rabbit lamellar keratectomy model by suppressing myofibroblast activation [125], and collagen/multi-arm PEG hydrogels served as efficient MSC delivery platforms that improved corneal clarity and downregulated stromal vimentin [126]. Consistency of source material and processing remains a key translational requirement.

Polysaccharides such as HA, alginate, and chitosan carry anionic/cationic groups that confer high water retention, mucoadhesion, and enzymatic degradability [[127], [128], [129]]. A dendrimer–HA hydrogel bearing dendrimer-dexamethasone (D-Dex) alleviated inflammation, improved clarity, and inhibited corneal neovascularization [58]. Chitosan's cationic mucoadhesion and antimicrobial activity enhance ocular delivery, chitosan-coated or -modified carriers—including CaP particles that prolong precorneal retention, cerium nanocapsules that increase corneal permeability 43-fold, nanoparticles (NPs) that double tetrahydropalmatine bioavailability, chitosan-modified PCL NPs (DRZ-CS-PCL-NPs) that improve corneal adhesion and permeability, and pilocarpine-loaded systems that normalize IOP within 4 h in rabbits [[130], [131], [132], [133], [134], [135]]. Although multiple studies report low acute toxicity [136], long-term ocular safety, degradation pathways, and degradation-product biocompatibility require further evaluation; cationic charge may pose cytotoxicity risks to corneal/conjunctival tissues with prolonged exposure [130].

#### 4.2.2 Synthetic polymers

**Fig. 10:**
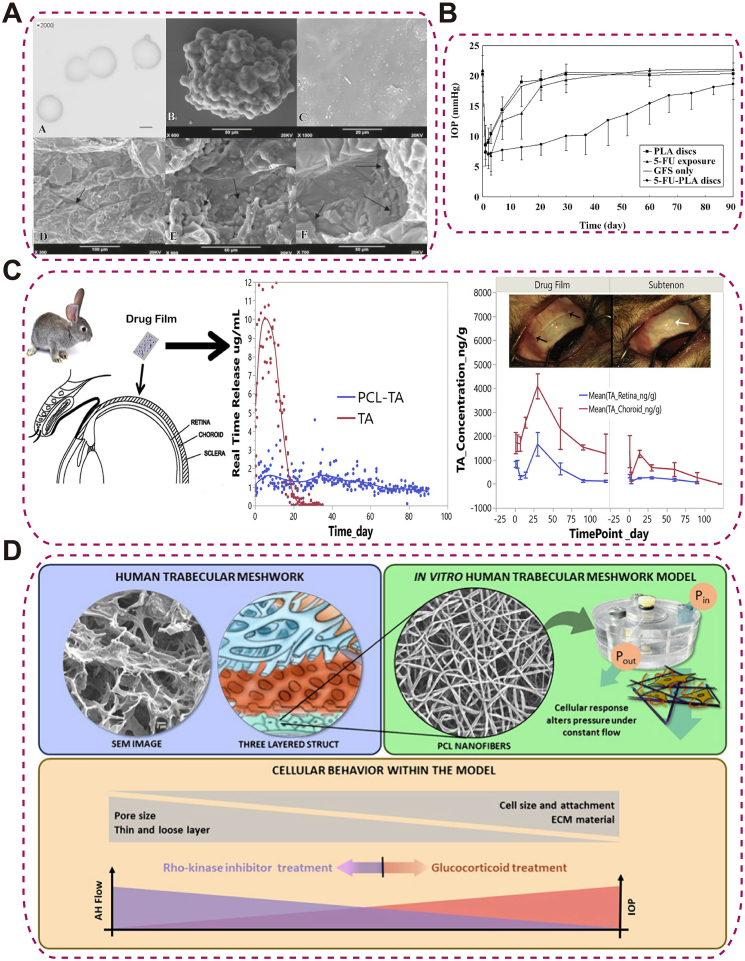
Synthetic Polymeric drug delivery systems. (A) External morphology of 5-FU-loaded PLA drug system (5-FU-PLA-DS) during in vitro release; (B) Postoperative IOP reduction in eyes treated with 5-FU-PLA-DS compared with controls, with significant differences from days 7–83 [139], copyright 2008, Acta Pharmacologica Sinica (A–B); (C) Multilayered PCL episcleral drug film enabling targeted delivery and controlled release [140], copyright 2016, Acta Biomaterialia; (D) Nanofibrous PCL-based human trabecular meshwork scaffold for aqueous humor outflow studies [142], copyright 2023, ACS Biomaterials Science and Engineering.

PLA is an aliphatic polyester that hydrolyzes to lactic acid; MW, stereochemistry/crystallinity, end-group, and geometry/porosity tune degradation and diffusion, while particle size and drug–polymer interactions control burst vs sustained release—enabling controlled ocular dosing. PLA has been extensively studied as a biodegradable sustained-release carrier in ophthalmology. For example, PLA microspheres have been applied for intravitreal delivery of doxorubicin to treat proliferative vitreoretinopathy (PVR), enabling sustained drug release and effectively inhibiting abnormal cell proliferation, while exhibiting no ocular toxicity and fully degrading within six weeks [137]. In retinal disease models, subconjunctivally injected budesonide-loaded PLA NPs/microparticles maintain therapeutic retinal concentrations and exert antiangiogenic effects by suppressing VEGF expression [138]. In glaucoma surgery, subconjunctival implantation of 5-fluorouracil (5-FU)-loaded PLA microspheres enables drug release for up to 91 days, significantly prolonging bleb survival, lowering IOP, and minimizing corneal toxicity compared with traditional drug administration [139] (Fig. 10A–B). However, PLA exhibits brittleness and limited thermal stability, which restrict its long-term mechanical performance in biomedical applications.

PCL is a biodegradable, semicrystalline polyester. Semicrystalline PCL (Tg ∼ −60 °C) degrades slowly over months–years; crystallinity/MW and geometry/porosity tune release, while hydrophobicity limits water uptake. PCL has demonstrated considerable potential in ophthalmic applications as a multifunctional drug delivery platform and a biomimetic scaffold. Sun et al. [140] designed a multilayered PCL scleral drug film loaded with triamcinolone acetonide (TA) for sustained release in chronic vitreoretinal diseases. In rabbit models, this system increased the area under the concentration-time curve (AUC) in the choroid and retina by 5.6-fold and 3.4-fold, respectively, with drug retention lasting up to 86 days (Fig. 10C). Kim et al. [141] developed an intraocular PCL implant capable of delivering the glaucoma medication DE-117 at a zero-order release rate (0.5 μg/day) for six months, achieving consistent distribution in ocular tissues such as the iris and ciliary body. Beyond drug delivery, PCL has also shown promise in disease modeling. Bikuna-Izagirre et al. [142] utilized PCL nanofiber scaffolds to construct a functional artificial trabecular meshwork, providing an innovative platform for studying glaucoma pathophysiology and drug screening (Fig. 10D). Nevertheless, the slow degradation rate and hydrophobicity of PCL can impede timely resorption and cell–material interactions.

### 4.3 Other bioactive composite materials

Second-generation composite biomaterials have demonstrated significant advantages in ophthalmology. Through organic–inorganic hybridization or polymer-assisted modification, these materials overcome the limitations of single-phase systems while optimizing mechanical strength, degradation kinetics, and functional integration.

**Fig. 11:**
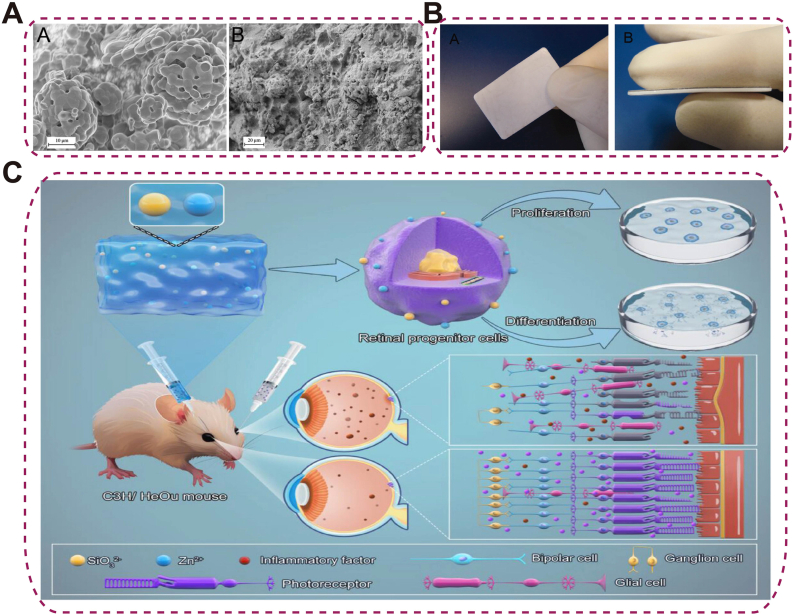
Composite and hybrid biomaterials for ocular applications. (A) High-resolution SEM images of porous β-TCP particles and their homogeneous distribution (30 vol%) within PTMC matrix; (B) Images of the prepared PTMC/β-TCP composite and the laminated PTMC/β-TCP composite sheets [143], copyright 2012, Journal of Biomedical Materials Research Part B (A–B); (C) Zinc silicate bioceramics enhancing retinal progenitor cell activation for functional restoration in retinal degenerative diseases [144], copyright 2025, VIEW.

For example, poly(trimethylene carbonate) (PTMC)/β-TCP laminated composites improve the flexural modulus to 64 MPa with 30 % β-TCP content, meeting structural requirements while avoiding acidic degradation typical of traditional polymers [143] (Fig. 11A–B). In retinal degenerative diseases, zinc silicate composite hydrogels promote neural regeneration by mimicking the ECM and activating AKT/ERK and Tiam2-Rac pathways. Zinc and silicon ions synergistically drive retinal progenitor cell proliferation and differentiation into photoreceptors while suppressing glial scarring [144] (Fig. 11C). However, mismatched degradation rates, weak interfacial bonding, and complex fabrication processes can limit their mechanical integrity and reproducibility.

Despite substantial progress, second-generation composite materials still face limitations, including insufficient biological modulation and a lack of dynamic responsiveness. These challenges are driving the exploration of third-generation smart composite materials that integrate stimulus-responsive components (e.g., thermosensitive or photosensitive polymers), ECM-mimicking features, or bioactive agents to enable spatiotemporally controlled tissue regeneration and cell–material interactions—advancing ophthalmic medicine toward precision and personalization.

## 5 Actively interactive smart ophthalmic biomaterials (Third-generation)

Third-generation biomaterials are defined as stimuli-responsive systems that passively adapt to changes in the ocular microenvironment by altering their structure or therapeutic output when exposed to specific cues. This generation shifted materials from static structural or merely biofunctional constructs toward platforms that undergo controlled physicochemical changes—such as swelling, degradation or triggered release—upon exposure to signals (pH, reactive oxygen species, enzymes, temperature, or light). Such designs enable spatiotemporal control of dosing but do not perform onboard decision-making or bidirectional modulation of tissue microenvironment, distinctions that separate them from emerging fourth-generation closed-loop platforms. In this review, “smart” denotes active interaction. We distinguish two pathways: responsive therapeutics, where materials sense ocular cues (chemical, thermal, mechanical) and modulate drug/gene release or local actuation; and functional replacement, where bioelectronic materials replace or augment lost photoreceptor function and interface with neural circuits.

### 5.1 Smart therapeutics (drug delivery)

Overcoming the unique anatomical and physiological barriers of the eye remains one of the most critical challenges in ophthalmic therapy. Smart biomaterials provide advanced delivery platforms that not only enhance intraocular drug bioavailability and prolong residence time, but also enable environment-responsive, targeted, and adaptive drug release [132,145,146]. These multifunctional systems integrate nanostructures—including polymeric NPs, liposomes, mesoporous carriers, hydrogels, and metal-organic frameworks—allowing precise modulation of therapeutic concentration, spatial distribution, and pharmacokinetics (Table 3). In parallel, smart materials have also emerged as highly promising gene delivery vehicles, offering improved targeting precision and nucleic acid payload capacity while minimizing immunogenicity and off-target risks. Building on these material innovations, diverse delivery platforms are being developed to address sustained release, responsive activation, barrier penetration, gene therapy, and targeted localization across a broad spectrum of ocular diseases.

**Table 3:** Summary of nanostructure-based smart drug delivery systems in ocular therapy.

Material Class	Representative Materials	Key Biological Functions & Clinical Advantages	Ocular Applications	Limitations
Hydrogels	GelMA, chitosan-based, collagen-based, SF hydrogels	Mimic ECM; promote cell adhesion/proliferation; sustained release; injectable and minimally invasive	Corneal repair; anti-scarring membranes; stem cell carriers	Mechanical fragility; rapid degradation without modification
Liposomes	PEGylated liposomes, cationic liposomes	Encapsulate hydrophilic/hydrophobic drugs; surface functionalization; sustained release	Corticosteroid delivery for uveitis	Limited loading for some drugs; leakage risk
Mesoporous carriers	Mesoporous silica NPs, bioactive glass NPs	High drug loading; tunable pore size; controlled release; easy modification	Anti-glaucoma drug delivery; orbital implant coatings	Long-term safety not fully established; Limited biodegradability
Metal-organic frameworks	ZIF-8, MIL-100, UiO-66	Ultra-high porosity for high drug loading; stimuli-responsive (e.g., pH, light) release	Photodynamic therapy; antioxidant and anti-inflammatory delivery; pH/ROS-responsive ocular drug and gene delivery	Stability issues in aqueous/biological environments; potential metal ion toxicity; synthesis complexity
Polymeric NPs	PLGA NPs, chitosan–carbome NPs,	Sustained and targeted delivery; tunable degradation	Anti-inflammatory agents; anti-VEGF; anti-glaucoma drugs	Possible burst release; immune response risk

#### 5.1.1 Environmentally responsive and advanced therapeutically integrated systems

Pathological cues in ocular microenvironments—such as altered pH, temperature, oxidative stress, and enzymatic activity—offer powerful triggers for smart biomaterials to achieve site-specific and temporally controlled drug release. Thermo-, pH-, ROS-, and enzyme-responsive systems have demonstrated marked improvements in ocular drug retention, bioavailability, and therapeutic precision. Building upon this foundation, multifunctional delivery platforms have been developed to co-deliver anti-inflammatory, anti-fibrotic, or anti-angiogenic agents, enabling synergistic effects and enhanced clinical efficacy. Moving further, next-generation biomaterials integrate biologically active components—such as nitric oxide (NO) donors, exosomes, oxygen generators, and immunomodulators—within responsive frameworks. These integrated systems transcend conventional delivery by actively participating in tissue repair, immune modulation, and microenvironmental reprogramming, offering programmable, multi-targeted strategies for complex ocular diseases.

##### 5.1.1.1 Thermo-responsive biomaterials

Thermo-responsive biomaterials leverage temperature-induced sol–gel transitions near physiological temperatures (30–37 °C) to enable minimally invasive intraocular administration and in situ depot formation [147]. At the lower critical solution temperature (LCST), dehydration/collapse of hydrophobic segments drives micellization and physical crosslinking (e.g., PNIPAM LCST ≈ 32 °C), forming an in situ gel that limits diffusion and the initial burst. The hydrophilic–hydrophobic block ratio, molecular weight, and polymer concentration set the gelation temperature (T_gel) and control mesh size/permeability and release kinetics. Polymers such as poly (N-isopropylacrylamide) (PNIPAM) [148], poloxamer [149,150], and PEG-PLGA copolymers [151] rapidly transition from liquid to gel following injection, thereby prolonging ocular residence time, stabilizing drug pharmacokinetics, and minimizing initial burst release.

**Fig. 12:**
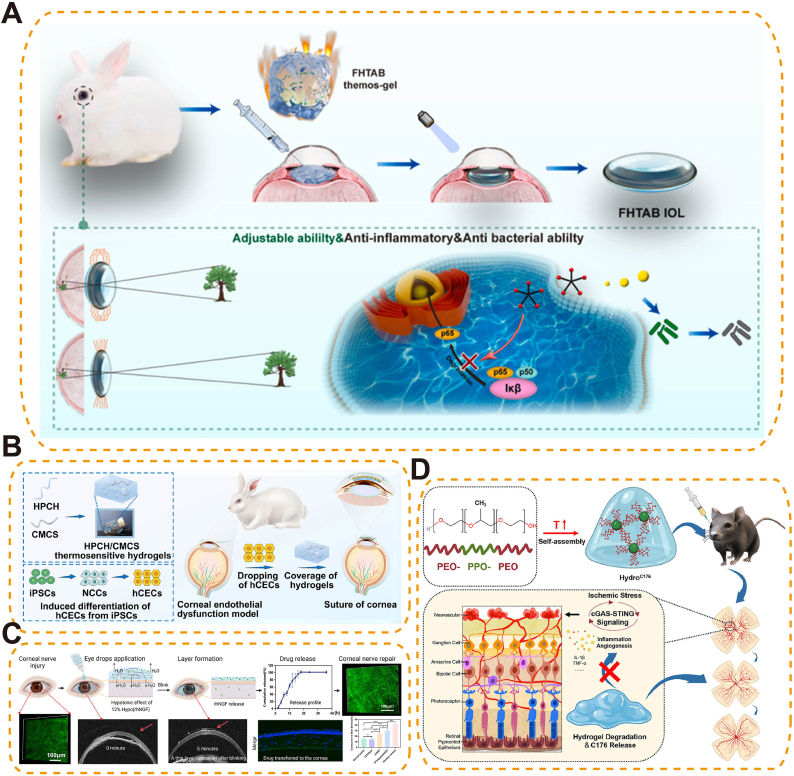
Thermosensitive hydrogels for ocular drug delivery systems. (A) Thermosensitive poloxamer-based antibacterial, anti-inflammatory, and photothermal conductive multifunctional hydrogel for injectable, in situ curable, and adjustable IOLs applications [152], copyright 2024, Bioactive Materials; (B) Thermosensitive hydrogel combined with iPSC-derived corneal endothelial cells for repairing rabbit corneal endothelial dysfunction [160], copyright 2025, Acta Biomaterialia; (C) Thermosensitive hypotonic PF127 hydrogel eye drops loaded with rhNGF for sustained release and corneal nerve regeneration in neurotrophic keratopathy [162], copyright 2025, Journal of Controlled Release; (D) PF127 thermosensitive hydrogel enabling sustained intraocular delivery of C-176 to block cGAS–STING signaling, reduce inflammation, and inhibit retinal neovascularization in OIR models [164].

Thermoresponsive drug delivery systems have been tailored for post-cataract care, where inflammation, fibrosis, and infection pose major challenges. For example, Qin et al. developed an injectable, NIR-responsive IOLs (FHTAB IOLs) that not only restored vision after cataract surgery but also released anti-inflammatory and antimicrobial agents, effectively preventing post-operative complications [152] (Fig. 12A) in a post-cataract surgery rabbit model. Similarly, a celastrol-loaded micelle-hydrogel (CMG) system suppressed TGF-β1-driven fibrosis, reducing PCO in rabbit models [153]. To mitigate post-operative endophthalmitis (POE), a thermosensitive chitosan/gelatin hydrogel (PAgel-LNPs) co-loaded with levofloxacin and prednisolone acetate was developed. This system gelled rapidly at ocular surface temperature, enabled dual-drug release for seven days, and significantly reduced inflammatory and bacterial burden in preclinical models [154].

In corneal diseases, thermo-responsive and integrated platforms enable both localized responsiveness and regenerative synergy. PNIPAM–chitosan-based hydrogels have been tailored for allergic conjunctivitis, achieving customizable rheology and multi-day release of ketotifen fumarate, with confirmed biocompatibility in both rats and human corneal epithelial cells (hCECs) [155]. Ferulic acid–loaded chitosan gels improved bioavailability and promoted epithelial repair in rabbit alkali burn models [156]. C-dots@Gel, a F127-based hydrogel loaded with ceria-like nanozymes, enhanced tear film stability, reduced oxidative stress, and accelerated corneal healing in a murine dry eye model, without ocular or systemic toxicity [157]. Building on these thermo-responsive formulations, recent systems incorporate multifunctional agents to address complex inflammatory and neurotrophic corneal conditions. A thiolated chitosan/β-glycerophosphate hydrogel (RDTG) sequentially released dipotassium glycyrrhizate and liposomal ginsenoside Rg3, accelerating healing and reducing stromal fibrosis in alkali burn mouse models [158]. Similarly, a poly(D,L-lactic acid) (PDLLA)-PEG-PDLLA hydrogel (DIC@Ava) co-delivering diclofenac micelles and Avastin® achieved dual-drug release over 19 days, significantly reducing neovascularization in a rabbit model compared with Avastin monotherapy [159]. In parallel, integrated systems enhance structural recovery by combining biomaterial responsiveness with biological therapies. For instance, induced pluripotent stem cells (iPSC)-derived hCECs embedded in hydroxypropyl chitosan/carboxymethyl chitosan (HPCH/CMCS) hydrogels restored corneal clarity in rabbit lamellar injury models [160] (Fig. 12B), while a thermosensitive chitosan hydrogel carrying iPSC–MSC-derived exosomes sustained miR-432-5p delivery to inhibit translocation-associated membrane protein 2 (TRAM2)-mediated collagen deposition, reducing stromal scarring in rats [161]. A hypotonic thermosensitive Pluronic F127 hydrogel incorporating recombinant human nerve growth factor (rhNGF) improved ocular retention and enabled sustained release, thereby promoting epithelial repair, enhancing corneal nerve regeneration, and restoring tear secretion in a mouse model of neurotrophic keratopathy, without affecting ocular morphology [162]-demonstrating the therapeutic advantages of integration, including improved bioavailability, prolonged activity, and synergistic tissue regeneration (Fig. 12C).

For vitreous and retina diseases, thermo-responsive hydrogels have enabled sustained drug delivery and therapeutic integration. A poly(β-butyrolactone-co-lactic acid)-PEG hydrogel (PBLA-PEG-PBLA) was developed for intravitreal ganciclovir (GCV) delivery, achieving 85 % release over 96 h via non-Fickian diffusion and significantly enhancing ocular bioavailability, half-life, and AUC in cytomegalovirus retinitis models compared to GCV solution [163]. In retinal inflammatory disorders, a Pluronic F127-based hydrogel encapsulating the STING inhibitor C-176 utilized inflammation-triggered in situ gelation to sustain drug release. This system effectively suppressed microglial activation and angiogenic inflammation in mouse retinal tissues [164] (Fig. 12D). In neovascular retinal diseases, such as wet AMD (wAMD), a Rab&Rapa-M@G hydrogel co-delivered rapamycin and ranibizumab in a sustained manner, restoring autophagic flux, reducing oxidative stress, and significantly suppressing choroidal neovascularization in the mouse model [165]. To simultaneously target inflammation and pathological neovascularization in the posterior segment, a thermo-responsive hydrogel based on poly(NIPAM-co-HEA/Furan) and poly(NIPAM-co-HEA/Maleimide) was developed to co-deliver anti-VEGF and dexamethasone. This in situ gelling system enabled tunable dual-drug release over 13–35 days, with confirmed cytocompatibility in RAW 264.7 and ARPE-19 cells, and demonstrated stable depot formation in ex vivo rabbit eyes [166].

In optic nerve injury, thermo-responsive hydrogels have enabled sustained neuroprotective delivery and combinatorial pathway targeting. A hydrogel co-delivering 3BDO and trichostatin A (TSA)—targeting the mTOR and HDAC pathways—was developed for intravitreal administration, achieving sustained release over four weeks. In a mouse optic nerve crush model, this dual-agent system promoted axon regeneration and significantly restored visual function [167]. To further improve outcomes in traumatic optic neuropathy (TON), a PLGA–PEG–PLGA thermogel was formulated for sustained intravitreal delivery of ciliary neurotrophic factor (CNTF) and TA. In a rat optic nerve crush model, this minimally invasive platform enhanced axonal regrowth and doubled retinal ganglion cell survival compared to untreated controls (31.05 % vs. 16.79 % at Day 28) [168]. Expanding on localized delivery strategies, a chitosan-based hydrogel co-loaded with CNTF and micelle-encapsulated FK506 was applied directly at the injury site. This system combined in situ gelation with sustained neurotrophic and immunomodulatory release, effectively reducing post-injury inflammation and glial scarring while promoting robust axonal regeneration [169].

##### 5.1.1.2 pH-responsive biomaterials

pH-responsive biomaterials leverage the acidic microenvironments commonly found in inflamed or infected ocular tissues to trigger site-specific drug release [170]. Protonation/deprotonation of weak polyelectrolytes (e.g., –COOH in polyacrylic acid; –NH_2_ in chitosan) modulates chain charge density and ionic crosslinking, driving swelling/deswelling or sol–gel transitions that enlarge mesh size and permeability to produce pH-dependent release; copolymer composition (effective pK_a_) and crosslink density set the transition pH and govern release kinetics. Polymers such as polyacrylic acid, chitosan, and cellulose derivatives undergo pH-induced sol–gel transitions or swelling, enabling precise control over drug kinetics. These systems are particularly effective in treating conditions like glaucoma, conjunctivitis, keratitis, and postoperative infections, where local acidosis provides a physiologically relevant trigger. Dual-responsive hydrogels that integrate both pH and temperature sensitivity further enhance therapeutic precision.

**Fig. 13:**
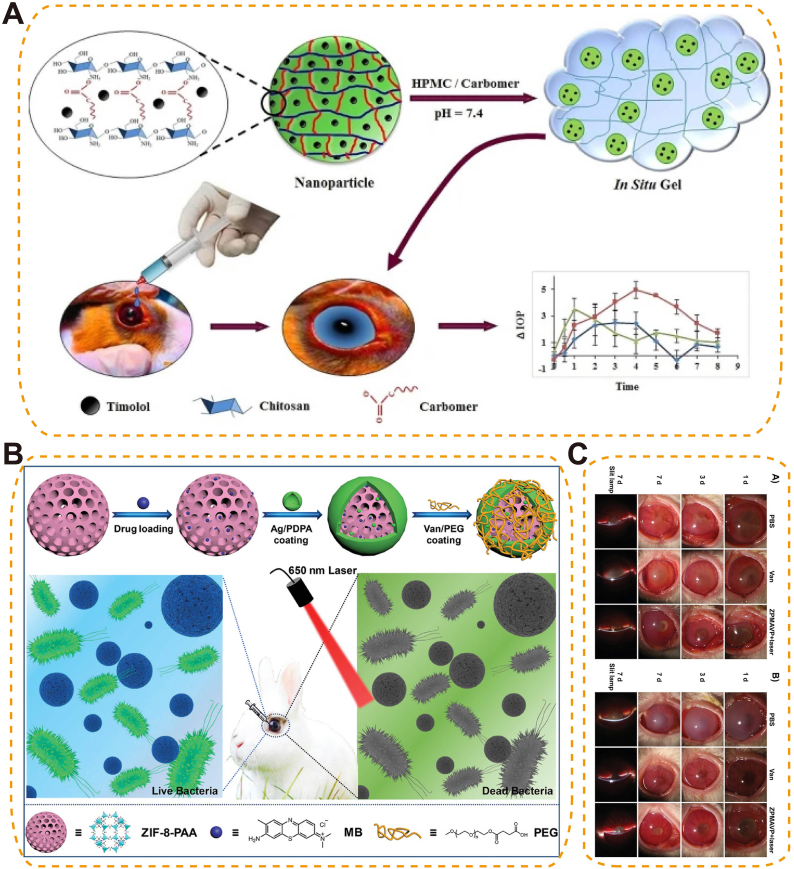
pH-responsive biomaterials for ocular drug delivery. (A) Chitosan/carbomer nanoparticle-loaded in situ gel providing sustained, pH-triggered release of timolol for improved glaucoma management [171], copyright 2025, Drug Delivery and Translational Research; (B) Zeolitic imidazolate framework (ZIF)-based pH-sensitive drug delivery system enabling synergistic chemotherapy and PDT for bacterial endophthalmitis; (C) Slit-lamp images showing therapeutic effects against *S. aureus* and MRSA endophthalmitis with PBS, vancomycin, or ZPMAVP NPs (zeolitic imidazolate framework–based, pH-responsive) plus laser irradiation (202 mW cm^−2^) over 1, 3, and 7 days [173], copyright 2019, Small (B–C).

In glaucoma therapy, the integration of timolol-loaded chitosan–carbomer NPs into a pH-responsive in situ gel enabled synergistic delivery. This hybrid system combined sustained drug release with mucoadhesion and pH-triggered viscosity enhancement, resulting in prolonged IOP reduction and improved ocular retention in rabbit models [171] (Fig. 13A).

To address superficial ocular infections and reduce the risk of antibiotic resistance, a pH-responsive multilayer film composed of methoxy poly(ethylene glycol)-poly(ε-caprolactone)-chitosan and montmorillonite (MPC/MMT) was engineered for the controlled delivery of triclosan. Under acidic conditions typical of inflamed ocular surfaces (pH 5.5), the film significantly enhanced drug release, effectively eradicating *S. aureus*, *E. coli*, and S. epidermidis without inducing cytotoxicity in human lens epithelial cells. This strategy offers a promising approach for postoperative or inflammation-associated surface infection control [172].

To tackle deep-seated intraocular infections such as biomaterial-associated endophthalmitis, a pH-sensitive nanoplatform (ZIF-8-PAA-MB@AgNPs@Van-PEG) was developed for targeted photodynamic antibacterial therapy. This hybrid system co-delivered methylene blue and in situ–formed silver NPs via a ZIF-8–polyacrylic acid scaffold, achieving infection-site–responsive release and synergistic antimicrobial effects [173] (Fig. 13B). In vitro and in vivo studies—including a murine endophthalmitis model—demonstrated potent bactericidal activity against *E. coli*, *S. aureus*, and MRSA, along with favorable retinal biocompatibility [173] (Fig. 13C). This system therefore holds promise as a synergistic delivery platform for targeted intraocular infection control.

##### 5.1.1.3 ROS-responsive biomaterials

ROS-responsive biomaterials harness the elevated oxidative stress in pathological ocular environments to enable controlled, stimulus-triggered drug release. In inflamed or neovascular tissues, increased ROS—especially H_2_O_2_—cleave ROS-labile linkers (e.g., thioketals, boronic esters, peroxalate esters) or oxidize thioethers to sulfoxides/sulfones, weakening hydrophobic domains or crosslinks and thereby increasing mesh size/permeability or inducing carrier disassembly for on-demand release; linker chemistry and crosslink density set the activation threshold and release kinetics. By incorporating ROS-cleavable linkages or redox-sensitive moieties, these materials ensure on-demand therapeutic delivery while concurrently mitigating oxidative damage. This strategy is particularly valuable in treating inflammation-driven and neovascular ocular diseases, where traditional sustained-release systems may fall short of environmental adaptability.

**Fig. 14:**
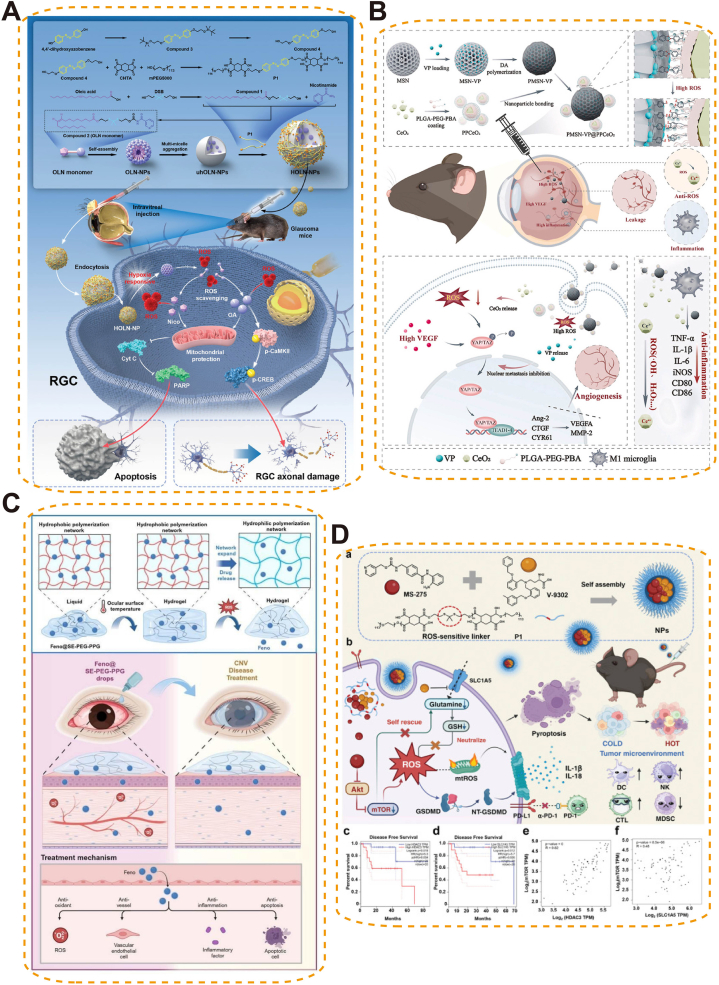
ROS-responsive biomaterials for ocular drug delivery. (A) Hypoxia/ROS dual-responsive HOLN-NPs releasing nicotinamide and oleanolic acid to protect retinal ganglion cells in glaucoma [174], copyright 2024, Advanced Science; (B) ROS-responsive mesoporous silica–PLGA-PEG composite NPs co-delivering verteporfin and cerium oxide for combined anti-angiogenic, antioxidant, and anti-inflammatory therapy in neovascular AMD [179], copyright 2025, Materials Today Bio; (C) ROS-responsive, selenium-containing thermosensitive SePEP hydrogel loaded with fenofibrate for sustained anti-oxidative, anti-inflammatory, and anti-angiogenic therapy in corneal neovascularization [177], copyright 2025, Journal of Controlled Release; (D) ROS-responsive NPs co-delivering histone deacetylase inhibitor (MS-275) and glutamine metabolism inhibitor (V-9302) to trigger a lethal ROS storm, induce pyroptosis, and enhance α-PD-1 immunotherapy efficacy in uveal melanoma [180], copyright 2024, Advanced Science.

In glaucoma, a dual-responsive nanoparticle system-hybrid oleanolic acid–nicotinamide NPs (HOLN-NPs) was designed to target both hypoxia and oxidative stress. Upon encountering pathological conditions, these NPs release nicotinamide and oleic acid to scavenge ROS and activate the CaMKII/CREB pathway, preserving mitochondrial function. In preclinical models, this approach effectively protected retinal ganglion cells and attenuated axonal damage [174] (Fig. 14A).

On the ocular surface, ROS-responsive hydrogels and microneedle systems have enabled minimally invasive and prolonged therapy. A detachable microneedle patch (catechol-engineered microneedle, CE-MN) enabled targeted delivery of cyclosporin A and epigallocatechin gallate (EGCG) to the lacrimal gland in Sjögren's syndrome–related dry eye (SSDE). By sensing local ROS levels, CE-MN facilitated on-demand release lasting over 48 h and reduced inflammation and immune infiltration in SSDE mouse models [175]. In corneal neovascularization, ROS-responsive delivery systems have addressed the dual challenge of inflammation and angiogenesis. A controlled-release nanogel (DEX@INHANGs) utilized thioketal linkers and host–guest cyclodextrin–adamantane interactions to achieve oxidative-stress-triggered dexamethasone release, with integrin β1-mediated corneal adhesion prolonging drug retention (>8 h). In vitro, this system demonstrated low cytotoxicity and anti-angiogenic effects; in chemically injured rabbits, once-daily application markedly reduced neovascularization while lowering steroid exposure [176]. Another example, Se-PEG-PPG (SePEP), a ROS-responsive thermosensitive hydrogel loaded with fenofibrate (Feno), adhered via selenium–mucin interactions and achieved ROS-triggered release. In murine models, SePEP-Feno eye drops significantly attenuated both neovascularization and inflammatory responses [177] (Fig. 14C).

In posterior segment diseases, ROS-responsive nanomaterials provide a foundation for precise, environment-triggered therapy. At the basic level, such systems exploit oxidative microenvironments to enable on-demand drug release; for instance, dexamethasone–curcumin co-loaded chitosan–poly(2,3-dimethylmaleic anhydride)-dextran–curcumin (CPDC) NPs were internalized by activated macrophages, suppressing inflammatory cytokines and activating the Nrf2/HO-1 axis, thereby achieving superior anti-inflammatory effects in uveitis while minimizing steroid exposure [178]. Building on this, multi-drug delivery platforms have been developed to simultaneously target angiogenesis, inflammation, and oxidative stress. A PMSN-VP@PPCeO_2_NP system, combining verteporfin and cerium oxide-modified polymers, enabled ROS-triggered photodynamic therapy (PDT) while exerting anti-angiogenic, anti-inflammatory, antioxidant, and anti-fibrotic effects in choroidal neovascularization models [179] (Fig. 14B). Advancing toward integrated bioactive systems, a nanoparticle formulation co-delivering the epigenetic agent MS-275 and glutamine metabolism inhibitor V-9302 synergistically elevated ROS and depleted glutathione to induce pyroptosis in uveal melanoma, while enhancing anti-PD-1 immunotherapy by converting immunologically “cold” tumors into “hot” [180] (Fig. 14D). In parallel, a mitochondria-targeted nanoparticle incorporating pH/ROS dual-responsiveness and chondroitin sulfate-mannose (CAS-Man) conjugates enabled controlled lutein delivery, reduced mitochondrial oxidative damage, and preserved retinal function in degeneration models [181]. This pH/ROS dual-responsive design exemplifies the advancement of smart delivery systems toward enhanced spatiotemporal control, enabling precise activation under pathological conditions and improved therapeutic specificity in retinal diseases.

##### 5.1.1.4 Photothermal/photodynamic-responsive biomaterials

Photothermal/photodynamic responsive biomaterials enable precise, non-invasive control via light activation for targeted ocular therapy. Under selected wavelengths, photothermal absorbers (e.g., Au nanorods, polydopamine, indocyanine green) generate localized heat to soften/open thermosensitive matrices or accelerate diffusion, while photosensitizers produce ^1^O_2_/ROS that cleave redox-labile linkers and weaken hydrophobic domains—both routes yielding on-demand release. The absorber/PS geometry and loading (aspect ratio, size, concentration) together with irradiation parameters (wavelength, fluence, pulse/continuous time) set the activation threshold and release rate; matrix composition/crosslink density governs mesh expansion and payload permeability. Functionally, these systems can amplify antibacterial, anti-angiogenic, or anti-tumor effects while minimizing systemic exposure and enabling spatial selectivity (e.g., NIR-triggered intravitreal depots or surface gels).

**Fig. 15:**
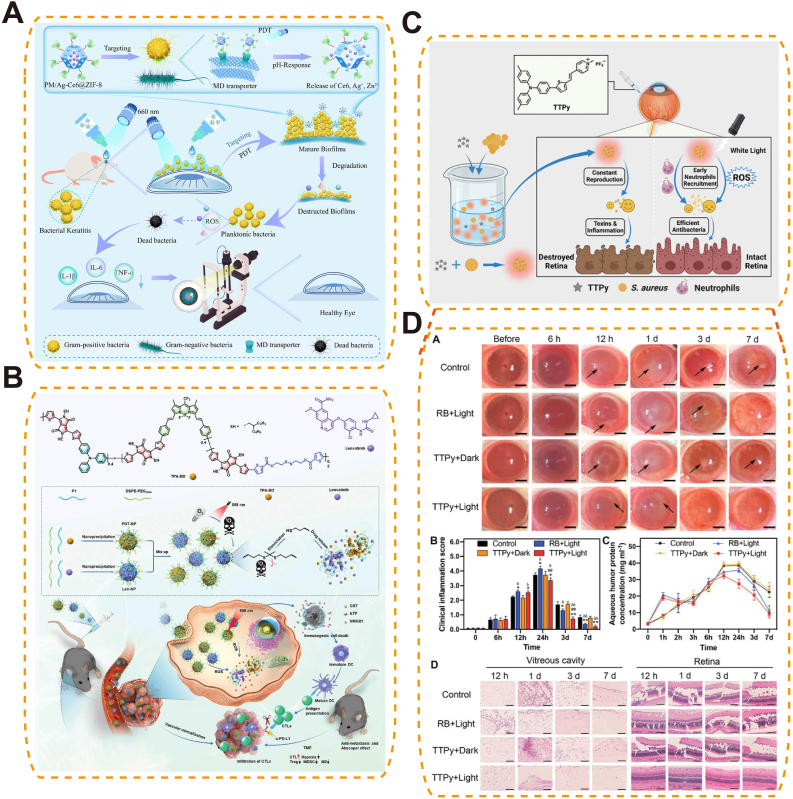
Photothermal/photodynamic-responsive biomaterials for ocular drug delivery. (A) Maltodextrin-modified metal–organic framework (MOF) nano-antibacterial system (PM/Ag-Ce6@ZIF-8) enabling bacterial targeting via maltodextrin transport pathway, silver nanoparticle-enhanced PDT, and potent biofilm inhibition for bacterial keratitis treatment [182], copyright 2025, Journal of Controlled Release; (B) NIR-II fluorescent biodegradable polymer NPs (Combo-NP) delivering lenvatinib to normalize tumor vasculature, enhance PDT efficacy, and combined with α-PD-L1 for photodynamic–immunotherapy and inhibition of cancer metastasis [187], copyright 2023, Advanced Science; (C) TTPy-based photodynamic antibacterial agent capable of rapid bacterial discrimination and efficient ROS generation under white light, triggering early immune activation to protect the retina from toxin- and inflammation-induced damage; (D) In vivo antibacterial efficacy of TTPy in a rat endophthalmitis model, demonstrating reduced ocular inflammation, decreased aqueous humor protein levels, and preserved vitreous and retinal structures compared with controls [184], copyright 2022, Advanced Science (C–D).

As light-responsive carriers, several nanoplatforms have achieved selective antibacterial effects in keratitis and related ocular infections. For example, pH-sensitive ZIF-8-based nanoplatforms (PM/Ag-Ce6@ZIF-8) [182] (Fig. 15A) and matrix metalloproteinase-sensitive supramolecular NPs (MMP-S NPs) have achieved infection-specific accumulation and activation in *Staphylococcus aureus* and *P. aeruginosa* keratitis models, respectively [183]. The acidic or MMP-rich microenvironments of infected corneas triggered carrier disassembly, exposing the photosensitizer chlorin e6 (Ce6) and enabling light-induced ROS generation for targeted bacterial ablation and effectively reducing inflammation and corneal damage [182,183]. Building on this strategy, a dual-functional nanoplatform (PPDL NPs) integrated catalase-like platinum NPs with dexamethasone to simultaneously boost ROS production through oxygen generation and mitigate inflammatory responses, resulting in effective bacterial clearance and biofilm elimination in *S. aureus* keratitis [183]. Similar photodynamic mechanisms have been extended to bacterial endophthalmitis, where a cationic aggregation-induced emission luminogen (TTPy) demonstrated selective bacterial targeting, potent ROS generation, and superior vision preservation compared with traditional photosensitizers such as Rose Bengal [184] (Fig. 15C and D). More advanced platforms integrate multiple bioactive mechanisms: A multifunctional near-infrared (NIR, 808 nm) responsive nanoplatform (UCNANs) integrated PDT with NO-mediated bactericidal action and anti-inflammatory effects. The system employed upconversion NPs (UCNPs) to convert NIR light into visible wavelengths, triggering the simultaneous release of ROS and NO. These reactive species synergistically generated peroxynitrite (ONOO^−^), enhancing bacterial killing and biofilm disruption. In a *Pseudomonas aeruginosa* keratitis mouse model, UCNANs significantly reduced corneal inflammation and biofilm burden, while downregulating inflammatory mediators such as TLR2 and TNF-α [185].

In uveal melanoma, photodynamic immunotherapy has been advanced through bioactive-integrated nanoplatforms that combine tumor-targeted delivery with immune activation. NPPDT-56MESS, a nanocomplex formed by electrostatic coupling of a photodynamic polymer (P1) and a cationic platinum(II) agent (56MESS), disassembles upon 808 nm laser irradiation to release ROS and 56MESS. This induces DNA and mitochondrial damage, elevates cytoplasmic double-stranded DNA levels, and activates the cGAS–STING pathway, triggering potent anti-tumor immunity and immune memory. In subcutaneous uveal melanoma models, it significantly suppressed tumor growth and prolonged survival [186]. To overcome the limitations of PDT imposed by tumor hypoxia and immunotherapy resistance, a multifunctional nanoplatform (Combo-NP) was developed by integrating a disulfide-containing pseudo-conjugated polymer (TPA-BD) with the vascular inhibitor Lenvatinib. This NIR fluorescent system enabled light-triggered ROS generation for tumor ablation, while simultaneously inducing immunogenic cell death to promote cytotoxic T cell infiltration. Moreover, vascular normalization improved intratumoral oxygenation and enhanced PDT efficacy. In uveal melanoma models, Combo-NP combined with anti–PD-L1 therapy significantly suppressed tumor growth and metastasis, exemplifying a photodynamically responsive and bioactive-integrated platform for synergistic tumor immunotherapy [187] (Fig. 15B).

##### 5.1.1.5 Enzyme-responsive systems

Enzyme-responsive nanomaterials leverage disease-associated enzymatic activity to achieve site-specific and temporally controlled drug delivery in ocular disorders. Specific enzyme–substrate pairs—e.g., MMP-cleavable peptides such as GPQGIWGQ (cleaved between G and I), glycosidic linkages for lysozyme, and ester linkages for esterases—are embedded in carriers so that enzymatic cleavage breaks crosslinks or hydrophobic domains, increasing permeability or inducing disassembly and thus on-demand drug release; substrate density and crosslinking dictate the activation threshold and kinetics.

**Fig. 16:**
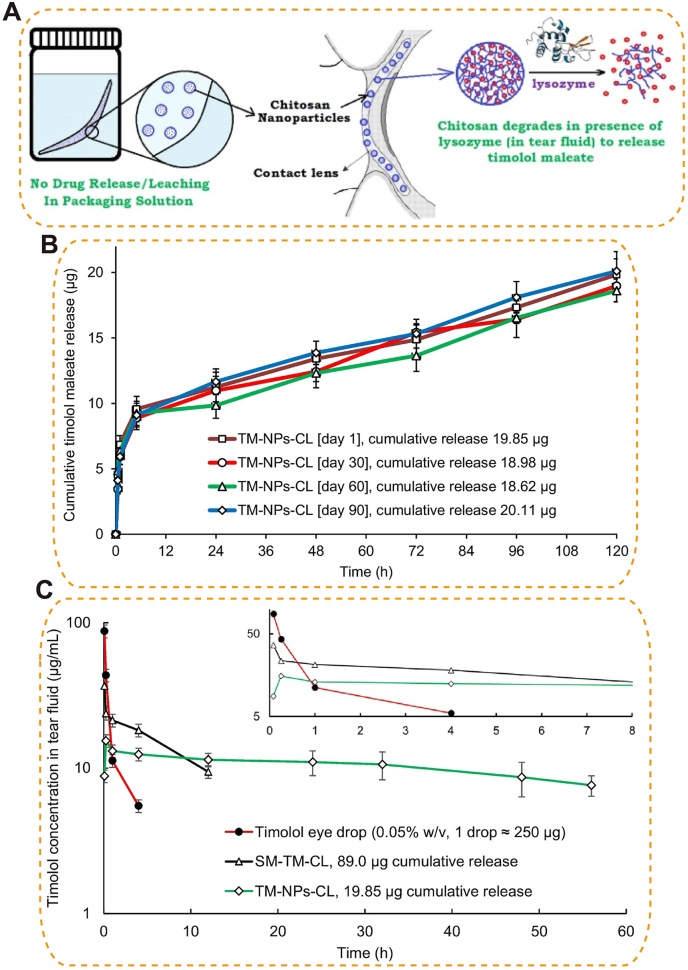
Enzyme-triggered drug-releasing CLs for glaucoma therapy. (A) Chitosan nanoparticle–laden CLs enabling enzyme-triggered, sustained release of timolol maleate for IOP control; (B) In vitro drug release profile showing stable, controlled release over three months of wet storage; (C) In vivo release kinetics in a rabbit model, demonstrating prolonged ocular drug delivery compared with conventional eye drops [189] copyright 2024, Drug Delivery and Translational Research (A–C).

MMP-sensitive nanocarriers have been widely employed to release anti-inflammatory or anti-fibrotic agents at inflammation or injury sites, thereby minimizing off-target toxicity and enhancing therapeutic efficacy [188]. Additionally, lysozyme-responsive hydrogel systems, such as nanoparticle-embedded CLs or in situ gelling ocular formulations, undergo enzymatic degradation in the tear film to achieve controlled drug release and prolonged precorneal retention, offering minimally invasive and long-acting ocular delivery options [189,190] (Fig. 16A–C). Research on enzyme-responsive materials in ophthalmology remains comparatively limited. Key factors include the spatiotemporal variability of ocular enzyme levels, the ubiquity of many enzymes (with off-target risks), and outstanding practical issues in sterilization-tolerant formulations and standardized in-vivo cleavage-to-release assays.

##### 5.1.1.6 Mechanical stimulus–responsive smart ophthalmic biomaterials

Mechanical stimulus–responsive smart ophthalmic biomaterials enable self-activated therapy by transducing ocular deformation (blink shear, IOP oscillations, axial stretch)—including sustained scleral strain in high myopia—into targeted action. They tightly couple structure and mechanism: oriented, high-β-phase PVDF/PVDF-TrFE films generate piezoelectric fields under strain to direct cell behavior and ECM remodeling; flexo/triboelectric interfaces on soft substrates accumulate charge with curvature or friction to gate local stimulation; strain-tunable meshes/microvalves and mechanolabile networks enlarge mesh size or open flow paths once stress exceeds a preset threshold, producing pulsatile drug release. Performance is set by β-phase fraction/orientation (piezoelectric coefficient), modulus matching/adhesion (mechanical coupling and comfort), and geometry/porosity/aperture design (activation threshold, permeability, flow rate), while labile-segment placement/density controls reversibility and release kinetics.

A representative design is the biomimetic piezoelectric patch (BPP@PVDF), inspired by the electric eel's capability to generate bioelectric fields. This construct integrates a bovine pericardium (BPP) scaffold with a piezoelectric polyvinylidene fluoride (PVDF) film. Under scleral deformation, the PVDF layer undergoes piezoelectric conversion, generating localized electrical fields that promote fibroblast adhesion, upregulate COL1A1 expression, and guide collagen fibril alignment—thereby restoring ECM density and tensile strength [191].

In vitro, electrical stimulation of human scleral fibroblasts significantly increased type I collagen production and improved fibril organization. In vivo, sub-Tenon's implantation in a rabbit model of myopia effectively reduced axial elongation, preserved posterior pole morphology, and enhanced scleral tensile properties without inducing chronic inflammation or fibrosis during extended follow-up. This illustrates the feasibility of using endogenous mechanical forces as a therapeutic activator in ocular disease management [191].

Beyond myopia, mechanical stimulus–responsive platforms hold potential in corneal stiffening for keratoconus, intraocular pressure–mediated neuroprotection in glaucoma, and strain-activated release from drug-eluting implants. Future developments may incorporate nanoscale piezoelectric fibers, mechanochromic indicators for feedback, and multi-modal hybrids combining mechanical, optical, and thermal responsiveness to realize precision-adaptive ocular therapies tailored to the evolving biomechanical state of the eye.

#### 5.1.2 Gene delivery systems based on smart materials

Smart biomaterials have emerged as next-generation platforms for non-viral gene delivery in ophthalmology, offering enhanced safety, programmability, and targeting specificity. Unlike conventional viral vectors—limited by immunogenicity, packaging size, and delivery constraints—smart nanocarriers exploit the eye's immune-privileged environment and enable precise, minimally invasive genetic modulation for inherited and acquired ocular diseases. Beyond expression, non-viral platforms increasingly support true gene editing in the retina, including CRISPR–Cas nuclease cutting, base editing for single-nucleotide correction, and prime editing for small insertions/deletions; editor choice, guide design, and delivery chemistry jointly determine efficiency, specificity, and durability. Editing strategies should be benchmarked by on-target efficiency, off-target profiling, and functional rescue (ERG amplitude, OCT structure, behavioral vision), with immune monitoring and longitudinal durability.

##### 5.1.2.1 Lipid-based delivery systems

**Fig. 17:**
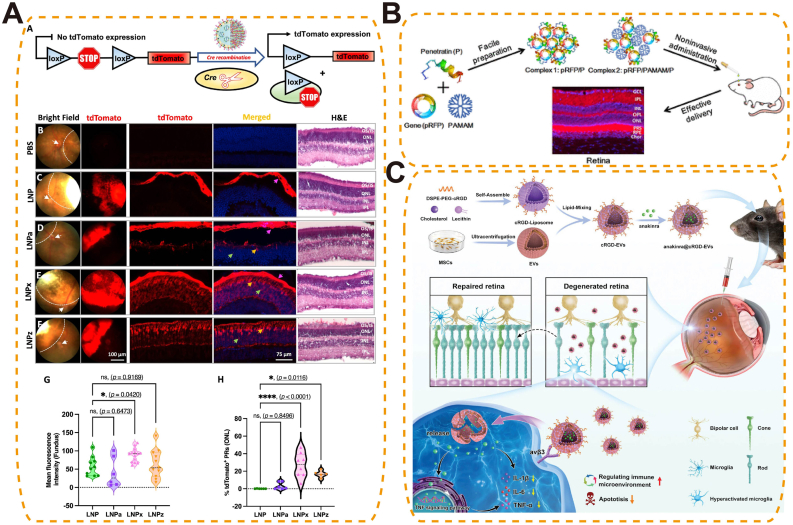
Smart Material-Based Gene Delivery Strategies for Ocular Therapy. (A) Lipid nanoparticle (LNP)-based gene delivery enabling Cre-dependent tdTomato expression for assessing retinal targeting efficiency across LNP variants in vivo [193], copyright 2023, Nature Communications; (B) Penetratin-functionalized peptide carrier for noninvasive retinal gene delivery [196], copyright 2016, ACS Applied Materials and Interfaces; (C) Cyclic RGD-modified EVs encapsulating anakinra for targeted retinal gene delivery and microenvironment modulation in retinal degeneration [198], copyright 2023, Small.

Lipid-based nanocarriers remain at the forefront of non-viral gene delivery. Ionizable lipids (pK_a_ ∼6–7) electrostatically pack and protect nucleic acids, remain neutral extracellularly, then protonate in endosomes to drive membrane fusion and endosomal escape; helper lipids/cholesterol set fusogenicity, PEG-lipid density controls stability/vitreous diffusion, and surface charge/ligands with particle size program retinal tropism and transport—enabling targeted mRNA/CRISPR delivery. For therapeutic editing, Peptide-modified lipid NPs (LNPs) were engineered to selectively target retinal photoreceptors, enabling delivery of mRNA or CRISPR–Cas9 across the neural retina. In nonhuman primates, this strategy achieved robust and cell-specific transgene expression in photoreceptor cells, opening therapeutic possibilities for inherited retinal dystrophies [192]. Further surface engineering with charged PEG-lipids has shown that LNP tropism can be tuned. For example, negatively charged LNPx and LNPz achieved up to 27 % photoreceptor transfection after subretinal injection, while positively charged LNPa favored retinal pigment epithelium (RPE) localization. LNPx carrying Cas9 mRNA enabled over 16 % genome editing in RPE [193] (Fig. 17A). By optimizing lipid composition, including removing cholesterol and modifying ionizable cores, LNPs have achieved organ-specific mRNA expression while minimizing off-target accumulation. This refined strategy enhances delivery precision and expands the potential of LNPs for targeted gene therapy, including ocular applications [194]. Editing performance should be reported alongside functional rescue metrics (ERG, OCT, behavior) and off-target assessments.

##### 5.1.2.2 Polymer- and protein-based platforms

Beyond lipids, synthetic polymer and protein-based vectors provide versatile scaffolds for gene delivery. Cationic/degradable polymers and protein/peptide vectors condense and protect nucleic acids via electrostatics; buffering capacity (proton-sponge), degradable linkers, and membrane-active domains enable endosomal escape and cytosolic release; ligand display/charge density/size program cell tropism and corneal/retinal transport—supporting targeted expression with reduced toxicity. Light-gated ionotropic glutamate receptor (LiGluR) constructs, chemically engineered to respond to optical cues, restored visual function in murine models of inherited blindness without inducing hepatotoxicity or immune activation [195]. A self-assembling nanocomplex developed by Pan et al. effectively penetrated the corneal barrier, enhanced gene expression, and suppressed VEGF activity in rat models, demonstrating promise for noninvasive treatment of AMD and DR [196] (Fig. 17B). As editor cargos diversify (CRISPR nucleases, base/prime editors), polymer/protein vectors can be tuned for payload size, intracellular trafficking, and release kinetics to balance efficiency and safety. At present, in vivo retinal editing data with polymer/protein vectors remain limited; systematic optimization and head-to-head evaluation against lipid and extracellular vesicles (EV) platforms therefore constitute a priority direction.

##### 5.1.2.3 Extracellular Vesicle–Based systems

Engineered EVs offer a virus-free, biocompatible platform for ocular gene and drug delivery. EVs’ lipid bilayer with native membrane proteins provides immune stealth and receptor-mediated uptake; ligand display (e.g., cyclic RGD) programs cell tropism (microglia/photoreceptors); biogenesis-based loading (e.g., ARRDC1/ESCRT-driven ARMMs for Cas cargos, CD63/ALIX or RNA-binding adaptors for nucleic acids) and endosomal escape features enable efficient cytosolic delivery, while 50–200 nm size supports vitreous/subretinal transport. Arrestin domain-containing protein 1-mediated microvesicles (ARMMs), efficiently package CRISPR–Cas systems and achieve over 60 % targeted gene editing in retinal cells with minimal immunogenicity [197]. Meanwhile, MSC-derived EVs modified with cyclic arginine-glycine-aspartic acid (RGD) peptides and loaded with the IL-1 receptor antagonist anakinra selectively target hyperactivated microglia, suppress inflammation, and protect photoreceptors in retinal degeneration models [198] (Fig. 17C). These strategies highlight the therapeutic potential of engineered EVs in combining gene editing and immune modulation for retinal diseases. Translation will require standardized assays for EV cargo quantification, targeting specificity, and longitudinal safety.

### 5.2 Smart functional replacement (artificial retina)

Artificial retina systems represent a paradigm shift in vision restoration for patients with advanced retinal degeneration such as retinitis pigmentosa (RP) and AMD. These bioelectronic prostheses aim to replace degenerated photoreceptors by directly stimulating surviving inner retinal neurons with patterned electrical signals. At the heart of this technology lies the integration of microfabricated electronics, neural engineering, and smart materials [199,200] (Table 4).

**Table 4:** Representative artificial retinal prostheses currently available or under development.

Product Name	Manufacturer	Availability	Materials/Technical Characteristics	Indications	Technical Advantages and Clinical Value
Argus II	Second Sight Medical Products, USA	Commercially available since 2011	Comprises an extracorporeal camera, image processor, and retinal implant with 60 electrodes; wireless transmission of stimulation signals	End-stage retinal degenerative diseases, including RP and AMD	Enables patients to perceive points of light, distinguish object contours, and, in some cases, recognize large-font text; first artificial retina system approved for clinical use worldwide
Norcom Artificial Retina	Zhejiang Norcon Neuroelectronics Technology Co., China	In clinical development	Extracorporeal video collector, processor, and retinal implant; greyscale image processing and noise reduction to stimulate the residual optic nerve and evoke light perception	RP and AMD	Addresses domestic technology gaps; ongoing clinical trials aim to provide basic light perception and object recognition
128-Channel Retinal Function Prototype	Shenzhen Zhongke Xianxin Medical Technology Co., China	In preclinical/early clinical development	Integrated mixed-signal system-on-chip with power and data management; microsystems and biocompatible materials	Severe visual impairment	Designed for high-channel retinal stimulation; aims to improve spatial resolution and functional vision restoration

Nanostructured photosensitive microelectrode arrays pair high interfacial area/low impedance and high charge-injection capacity (Au–Ti nanowires, conductive polymers, soft organic semiconductors) with tissue-matched mechanics (kPa–MPa), enabling intimate retinal contact and stable neuron–electrode coupling; bandgap/Schottky interfaces and plasmonic effects set spectral responsivity and photo-to-current transduction, while surface nanotopography promotes cellular adhesion and limits gliosis.

**Fig. 18:**
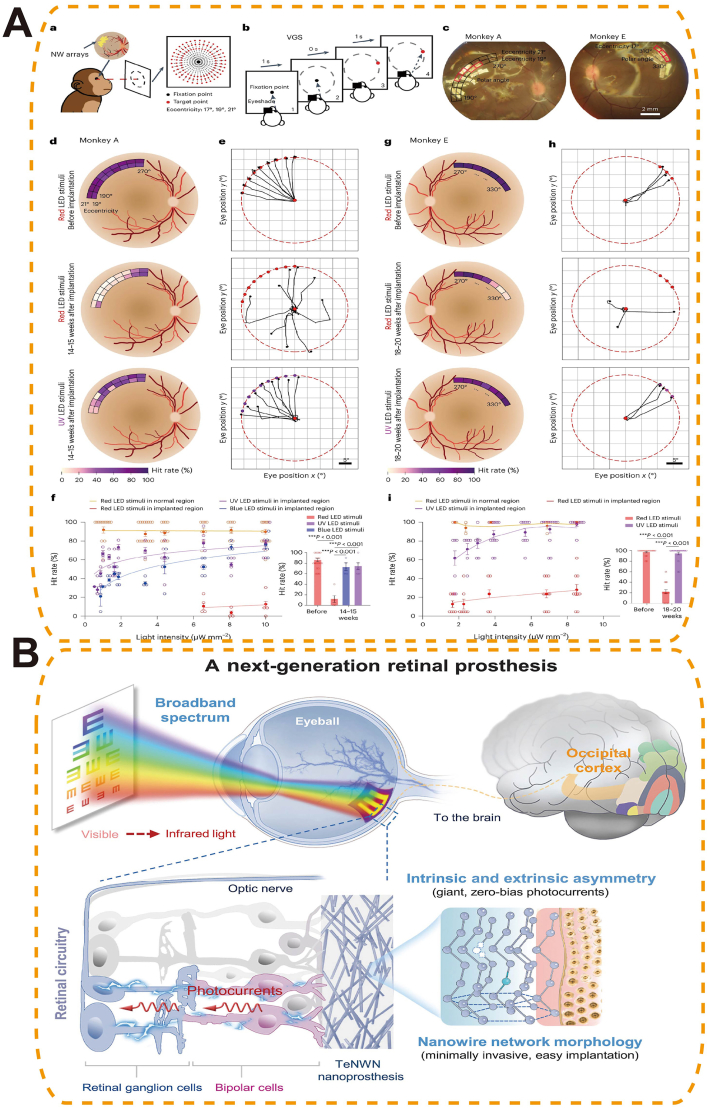
Artificial Retina Systems Based on Nanostructured Smart Materials. (A) Visual‐guided saccade task in primates with subretinal nanowire arrays, showing fundus images, light‐evoked responses, and long‐term functional integration [201], copyright 2024, Nature Biomedical Engineering; (B) Tellurium nanowire network prosthesis with broadband visible–NIR sensitivity, generating spontaneous photocurrents to restore vision while preserving native light perception [205], copyright 2025, Science.

Artificial retina systems based on third-generation smart materials typically consist of photosensitive microelectrode arrays fabricated from nanostructured materials—such as gold-titanium nanowires, conductive polymers, or soft organic semiconductors—that mimic the physical and electrochemical properties of native retinal tissue. These arrays are engineered for mechanical compliance, allowing intimate contact with the curved retinal surface without inducing chronic irritation or inflammation. In preclinical models of photoreceptor degeneration, subretinally implanted gold-nanoparticle-coated titania nanowire arrays achieved a spatial resolution of 77.5 μm and a temporal resolution of 3.92 Hz, enabling the detection of drifting gratings and flashing stimuli in blind mice with visual acuities of 0.3–0.4 cycles per degree. In nonhuman primates, the implants demonstrated long-term biostability (≥54 weeks), triggered visually guided saccades at low light thresholds (∼10 μW mm^−2^), and induced cortical plasticity, indicating functional integration with higher visual centers [201] (Fig. 18A). Complementing behavioral and cortical findings, implanted cohorts now report pattern-reversal VEP amplitude/latency as objective brain-level readouts, alongside simultaneous stimulate-and-record paradigms that enable closed-loop calibration of pulse amplitude, width, and frequency to achieve target percepts with minimal charge [202]. Clinically, the wireless subretinal photovoltaic PRIMA system in geographic-atrophy AMD shows four-year safety with preserved natural peripheral vision and central prosthetic letter/word recognition; with magnification, visual-acuity gains up to +8 ETDRS lines have been reported [203]. On the engineering side, photovoltaic arrays with dynamic field confinement improve spatial selectivity within safe charge limits, supported by modeling and in-vitro/in-vivo measurements—indicating a pathway to higher prosthetic acuity under clinical constraints [204].

Emerging materials such as tellurium nanowire networks further expand functional capabilities by enabling broadband light responsiveness, including NIR vision. These nanowires exhibit spontaneous, high-magnitude photocurrents under zero-bias conditions due to intrinsic lattice asymmetries and interfacial effects, supporting visual perception under ultralow light intensities. Subretinal implantation in blind mice and nonhuman primates restored optokinetic reflexes and light-guided behaviors, while maintaining biocompatibility and avoiding bulky external hardware. Notably, infrared responsiveness was achieved without compromising native visible-light vision [205] (Fig. 18B).

### 5.3 Clinical performance and limitations

Third-generation systems add environment coupling and spatiotemporal control (thermo/pH/ROS/enzyme triggers; light-guided release), extend non-viral gene delivery and editing, and show eye–brain efficacy in artificial retinas (VEPs, saccades, early clinical data). Key limits are in-vivo trigger variability, sensor drift/fouling, and safety/manufacturing constraints (oxygen, heat/charge, optical clarity, sterilization of soft hybrids); enzyme-responsive platforms are least mature. Evidence is moderate to strong for thermogels, pH/ROS depots, and subretinal prostheses; emerging for enzyme-gated delivery and polymer/protein vectors for retinal editing. Priorities: standardize trigger to release and latency/dose-error metrics, integrate bounded-autonomy control with clinician override, co-design to meet oxygen/thermal/charge limits, and develop closed-loop calibration.

## 6 Future direction: Closed-loop and autonomous smart materials for ocular Therapy (Fourth-generation)

Fourth-generation ophthalmic biomaterials are defined as intelligent, self-regulating platforms designed to enable closed-loop therapeutic control by integrating environmental sensing, data processing, and feedback-controlled therapeutic responses. Unlike earlier reactive or stimulus-driven systems, these materials continuously monitor ocular microenvironments and autonomously adjust drug delivery or functional output in real time to achieve close-loop regulation [206,207]. Although current prototypes remain largely semi-closed—dependent on external triggers or preprogrammed feedback—they lay the conceptual and technological foundation for adaptive and precision ocular medicine.

### 6.1 Design principles and challenges of closed-loop controlled ophthalmic biomaterials

Fourth-generation smart materials transcend simple responsiveness by incorporating active feedback loops that mimic biological homeostasis. Closed-loop smart materials couple transducers (pressure/chemical/oxygen sensors), computing elements (threshold logic, low-power ICs/ML blocks), and actuators (drug microvalves, electrostimulation, mechano/thermogels) within soft, tissue-matched architectures; material chemistry/porous and interconnect design set biocompatibility and signal fidelity, while energy/communication modules (piezoelectric harvesters, inductive links, biofuel cells; antennas) determine autonomy and telemetry—together enabling sense→decide→act feedback in the eye. These materials integrate biosensors, logic processors, and actuators within a unified platform to detect intraocular changes—such as pressure spikes, cytokine surges, or hypoxia—and execute calibrated responses. Dynamic materials like piezoelectric nanogenerators convert ocular motion into power [208], while embedded AI circuits adjust drug dosing or electrical stimulation in real time [209]. Self-healing hydrogels [210], biomimetic polymers [211], and tissue-integrating structures [212] ensure chronic biocompatibility and seamless neural interfacing.

The development of closed-loop controlled biomaterials for ocular applications necessitates the careful balance of biocompatibility, functional integration, and long-term stability. Material selection must minimize immune responses and allow for controlled biodegradability, often through surface modifications that mimic native ECM components to enhance cellular compatibility. Miniaturization is critical for implantability, demanding that sensors, processors, and actuators be integrated into compact, flexible forms without sacrificing performance. Powering these devices remains a central challenge, prompting exploration of wireless energy transfer, biofuel cells, and motion-based energy harvesting [213,214]. Similarly, wireless communication technologies are vital for real-time data transmission and clinical oversight [215]. Ensuring system stability involves addressing enzymatic degradation, mechanical fatigue, and maintaining the long-term precision of biosensors and actuators. In parallel, robust control algorithms must be developed to accurately decode complex biological inputs and initiate timely, appropriate therapeutic responses [216]. Together, these design principles highlight both the promise and complexity of engineering truly autonomous ocular biomaterials.

### 6.2 Recent advances in closed-loop controlled ophthalmic biomaterials

#### 6.2.1 Core biomaterials enabling autonomous ocular interfaces

Next-generation ocular biomaterials are increasingly characterized by their ability to support multimodal sensing, autonomous therapeutic actuation, and long-term integration with ocular tissues. Zwitterionic networks present charge-balanced hydration layers that suppress protein/cell fouling (stable interfaces); high–surface-area conductors (Au nanowires, graphene) provide low-impedance transduction for sensing/stimulation; and ECM-mimetic motifs (recombinant collagen, laminin/RGD cues) drive epithelial adhesion and immune tolerance—together enabling sense→decide→act autonomous ocular interfaces. Zwitterionic hydrogels have been applied to CLs and ocular surface coatings to reduce protein fouling and enhance ocular tolerance, offering improved biocompatibility and interface stability over conventional hydrogels [217]. Conductive nanoscale materials—such as gold nanowires [201] and graphene derivatives [[30], [31], [32]]—have been integrated into smart CLs and subretinal implants to enable real-time monitoring of IOP, tear glucose, and retinal electrical activity. These platforms allow transduction of physiological stimuli into electrical or optical signals, enabling feedback-driven actuation such as drug release or neural stimulation. Biologically derived materials, including recombinant collagen [218] and laminin-mimetic peptides [219], have been employed in engineered corneal scaffolds to enhance epithelial regeneration and immune tolerance. When combined with advanced fabrication strategies such as RGD-functionalized surface patterning and iPSC-laden GelMA hydrogels [220], these systems may support semi-living ocular implants that mimic tissue homeostasis and enable autonomous therapeutic responses. Collectively, these clinically oriented innovations demonstrate the emergence of truly closed-loop, fourth-generation ocular biomaterials (Table 5).

**Table 5:** Representative materials enabling fourth-generation closed-loop ocular systems and their key performance characteristics.

Material/System	Category	Key Performance/Properties	Representative Application
Zwitterionic hydrogels	Biocompatibility & Structural Support Materials	High antifouling, reduces protein adsorption, enhances ocular tolerance	CLs, ocular surface coatings
Recombinant collagen and laminin-mimetic peptides	Biocompatibility & Structural Support Materials	Promote epithelial regeneration, immune tolerance	Engineered corneal scaffolds
iPSC-laden GelMA hydrogels	Biocompatibility & Structural Support Materials	Supports semi-living implants, mimics tissue homeostasis	Advanced ocular implants
Conductive nanoscale materials (gold nanowires and graphene derivatives)	Sensing & Monitoring Materials; Energy Harvesting & Powering Materials	High electrical conductivity, flexible, biocompatible, transduces physiological stimuli into electrical/optical signals, can convert physiological signals into electrical energy	Smart CLs, subretinal implants
Battery-free sensors based on RFID or passive resonance	Sensing & Monitoring Materials; Energy Harvesting & Powering Materials	Ultrathin, flexible, wireless, continuous monitoring, harvests energy from external sources	Smart CLs, intraocular implants
Nanostructured biosensors	Sensing & Monitoring Materials	High sensitivity, integrates into hydrogels, noninvasive and continuous tear diagnostics	Hydrogel-based CL devices
Embedded ERG microelectrodes	Sensing & Monitoring Materials	High-resolution retinal electrophysiological signals	Smart CLs, retinal monitoring
Smart microneedles and in situ gelling hydrogels	Drug Delivery & Controlled Release Materials	Stimuli-responsive, spatiotemporal control, prolongs ocular residence time, reduces dosing frequency	Controlled ocular drug delivery
AI-controlled electroresponsive hydrogel reservoirs	Drug Delivery & Controlled Release Materials	Stimuli-responsive, AI-adaptive drug release	Smart CLs, closed-loop therapy
Non-invasive ultrasound emitters	Drug Delivery & Controlled Release Materials	Stimuli-responsive neuromodulation, non-invasive	Retinal neuromodulation
Frequency-encoded smart CLs materials	Data Transmission & Communication Materials	High-accuracy eye tracking, AI analysis of sequential eye movements	Eye–machine interaction, AR interfaces
Onboard or cloud-assisted logic units	Data Transmission & Communication Materials	AI-driven adaptive control, real-time decision making	Closed-loop ocular homeostasis

#### 6.2.2 Integrated Sensing–Actuation platforms for Closed-Loop Ocular Therapy

To achieve real-time, adaptive treatment, fourth-generation ocular systems integrate sensing and actuation modules that work in tandem to detect physiological changes and autonomously initiate therapeutic responses.

##### 6.2.2.1 Smart sensing interfaces

**Fig. 19:**
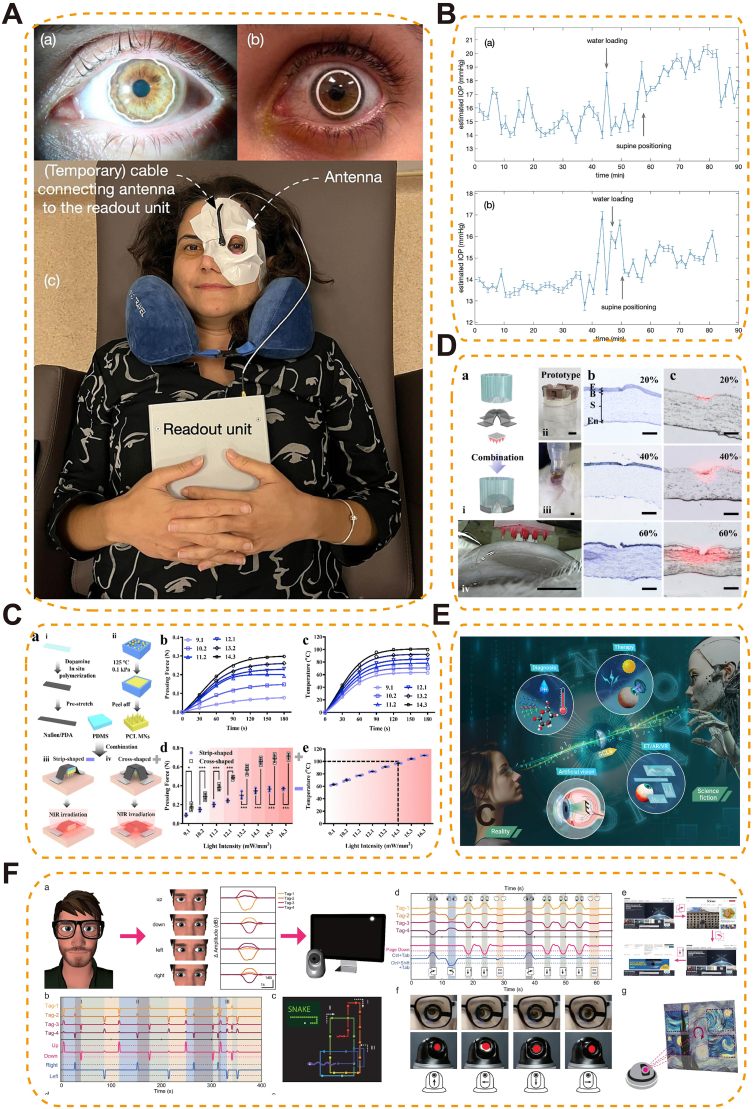
Integrated Sensing-Actuation Platforms for Closed-Loop Ocular Therapy. (A–B) Wireless smart CLs integrating IOP sensing and telemetry for continuous, non-invasive glaucoma monitoring in human subjects [222], copyright 2024, Contact Lens and Anterior Eye; (C) Light-responsive microneedle soft patch systems (MSPS) designed with shape-memory polymers and PCL microneedles, enabling NIR light-triggered mechanical actuation for adjustable penetration force and controlled drug release; (D) In vivo evaluation of MSPS in rabbit cornea demonstrating NIR-regulated microneedle penetration depths, minimal tissue damage, and enhanced diffusion of coated therapeutics [226], copyright 2023, Materials Horizons (C–D); (E) Multifunctional smart CLs combining diagnostics, therapy, and human–machine interaction for applications in artificial vision, AR/VR, and personalized ocular care [228], copyright 2023, Innovation (Camb); (F) Eye-machine interface platforms using real‐time eye‐movement signals to control virtual environments, webpages, and remote cameras, demonstrating expanded SCL-based interactive capabilities [232], copyright 2024, Nature Communications.

Battery-free sensors based on radiofrequency identification (RFID) or passive resonance have been embedded into smart CLs and intraocular implants to enable continuous, wireless monitoring of intraocular parameters [221,222] (Fig. 19A–B). LC resonators with serpentine metal traces on ultrathin elastomer/hydrogel substrates provide near-field power coupling and backscatter telemetry while preserving corneal conformability; nanostructured recognition/transduction layers (enzymes or aptamers with redox mediators; nanoporous electrodes) confer analyte selectivity and low-impedance signal amplification; hydrated polymer networks with high water content and mesh size enable rapid tear diffusion and continuous sampling—together supporting battery-free, multimodal monitoring. These ultrathin, flexible systems harvest energy from external sources and transmit data without disrupting ocular biomechanics. In parallel, nanostructured biosensors have been embedded in hydrogel-based devices, including CLs, to dynamically monitor tear composition, enabling noninvasive and continuous disease diagnostics [223]. Some advanced ocular platforms integrate multiple sensing modalities—such as oculometric data, IOP and ocular surface temperature—into a single soft interface, enabling more comprehensive, real-time assessment of ocular physiology [224,225].

##### 6.2.2.2 Advanced stimuli-responsive actuators

Smart microneedles and in situ gelling hydrogels have been developed to enable spatiotemporally controlled therapeutic release in response to ocular cues [226] (Fig. 19C–D). Microneedle geometry (length/tip radius/base angle) and biodegradable matrices (e.g., PLGA/PCL, polysaccharides) determine penetration force, depot formation, and burst vs sustained release; stimuli-responsive hydrogel chemistry (LCST/pH/enzyme/ROS) and crosslink density/mesh size set gel point, permeability, and release kinetics. In bioresponsive cell scaffolds, hypoxia/inflammation-inducible promoters and ECM-mimetic hydrogels couple pathological cues to on-demand factor secretion and integration, enabling spatiotemporally controlled ocular actuation. By integrating environmental responsiveness, these systems prolong ocular residence time and reduce dosing frequency, thereby improving treatment compliance and efficacy. Furthermore, bioresponsive scaffolds embedded with therapeutic cells are being investigated as regenerative platforms that activate repair processes in response to pathological signals like hypoxia or inflammation [227]. Although current systems lack full closed-loop feedback, they represent a critical step toward more intelligent ocular actuators.

Together, these sensing–actuation systems establish the functional backbone of closed-loop ocular therapies, enabling intelligent, self-regulated intervention that adapts dynamically to disease state and microenvironmental changes.

#### 6.2.3 Representative closed-loop ocular systems: smart CLs as a paradigm

Although no current ocular system fully embodies fourth-generation biomaterials—with real-time sensing, autonomous decision-making, and feedback-controlled therapeutic execution-smart CLs represent a compelling early-stage prototype. A soft, oxygen-permeable hydrogel chassis with curvature-matched mechanics hosts transparent conductors/microelectrodes and LC resonators/antennas for selective biosignal transduction and battery-free telemetry (input); patterned optics and frequency-encoded interconnects enable eye-motion parsing and user I/O (interface); and electroresponsive reservoirs/ultrasound emitters provide dose-controlled drug release or neuromodulation (actuation)—together supporting a closed-loop smart-CL paradigm.

At the input layer, smart CLs integrate multimodal biosensing capabilities, enabling continuous and noninvasive monitoring of IOP, tear biomarkers (e.g., glucose, cytokines), and notably, retinal electrophysiological signals via embedded ERG microelectrodes. Leveraging previously described battery‐free wireless sensing approaches, these systems achieve real‐time intraocular data acquisition without the need for onboard power, seamlessly integrating with soft, biocompatible ocular platforms. These features extend sensing beyond biochemical cues to the neural functional status of the retina, offering unprecedented real-time insight into ocular physiology and disease progression [228] (Fig. 19E).

Decision/processing layer (AI-assisted, off-lens). In current prototypes, data are off-loaded to near-eye or handheld processors (e.g., smartphone/earpiece) for analytics [229]. Lightweight algorithms—Kalman/AR models for drift correction and trend detection in IOP, supervised classifiers for tear-cytokine signatures, and ERG feature extraction (a-/b-wave amplitude, implicit time) for dysfunction screening—could generate control signals under clinician-defined limits [230,231]. Where appropriate, compact ML controllers (e.g., anomaly detection for IOP spikes, biomarker-gated microdosing policies) can be used to predict impending excursions and preemptively adjust dose; training remains supervised and safety-bounded, with human override.

At the interface layer, recent implementations of frequency-encoded smart CLs have demonstrated real-time, high-accuracy eye tracking using time-sequential signal analysis. By translating user-defined eye movements into command inputs, these systems enable intuitive control across both virtual and physical platforms—including games, web navigation, and robotic camera control, allowing for intuitive eye–machine interaction and immersive augmented reality (AR) overlays. These bidirectional interfaces enable real-time user feedback while forming the computational substrate necessary for adaptive logic [232] (Fig. 19F).

At the actuation layer, though still in development, emerging strategies include electroresponsive hydrogel reservoirs for stimulus-responsive drug delivery and non-invasive ultrasound emitters for retinal neuromodulation [41,233]. Coupled with the above edge/Cloud AI-assisted decision layer, these elements enable conditional execution (e.g., IOP band control, cytokine-threshold microdosing, ERG-informed stimulation titration) within safety envelopes.

Thus, smart CLs are evolving from passive diagnostics into integrated, neurobiologically informed feedback systems that sense, compute, and intervene within a self-contained platform. While fully on-lens autonomy is not yet realized, the AI-assisted architecture described here outlines a practical path toward closed-loop, safety-bounded operation for fourth-generation smart biomaterials.

### 6.3 Translational and technical challengess of closed-loop controlled ophthalmic biomaterials

Despite advances in smart ocular biomaterials, most current “smart” ocular materials remain at a passive or semi-closed-loop stage, lacking true self-regulatory capability. Functional integration is often limited, computational algorithms are immature, and energy management as well as long-term reliability remain critical constraints. For example, smart microneedles and in situ gelling hydrogels have been developed to provide spatiotemporally controlled drug release in response to ocular cues, but they cannot continuously and dynamically monitor physiological changes to adjust the release amount or rhythm [226]. Similarly, conductive nanoscale materials such as gold nanowires or graphene derivatives enable monitoring of intraocular pressure, tear glucose, or retinal electrical activity [[30], [31], [32]], but integration with autonomous actuators and adaptive AI-driven modulation is still lacking. These examples highlight the gap between current semi-closed-loop systems and fully autonomous fourth-generation ocular biomaterials, which would continuously sense physiological changes, interpret the data via embedded algorithms, and adjust therapeutic output to maintain ocular homeostasis.

Clinically, the translation of fourth-generation closed-loop ocular systems faces multiple intertwined challenges. Long-term biosafety — a key yet often underexplored issue in the development of autonomous ocular systems —remains a major bottleneck to clinical translation. Ensuring the chronic safety and functional stability of key components, including power supply, wireless communication, and miniaturized devices, continues to be a central concern. For wearable systems, the battery supply represents the primary safety concern, mainly due to risks of overheating or electrolyte leakage; yet the overall hazards remain low and are typically limited to surface irritation. In contrast, implantable devices require rigorous long-term validation, including cytotoxicity, genotoxicity, and chronic inflammation testing in large-animal models to ensure neural and retinal safety [208]. Wireless power systems must comply with specific absorption rate (SAR) limits and prevent localized tissue heating or electromagnetic interference [213,214], while encapsulation materials such as PDMS/SBS hybrid membranes offer protection against corrosion and leakage [215]. Nevertheless, degradation by-products (>6 nm) or fibrosis-induced encapsulation can still compromise long-term stability [212]. Miniaturization of electrodes introduces trade-offs among spatial resolution, impedance, and thermal noise, demanding careful optimization to prevent overstimulation or device fatigue [212]. Encouragingly, recent studies on nanocomposite ocular implants and battery-free smart contact lenses have demonstrated favorable biocompatibility, stable wireless performance, and the absence of corneal or retinal toxicity after long-term in vivo testing [211,[213], [214], [215]].

Beyond long-term biocompatibility, clinical translation faces additional critical hurdles. Individual physiological variability between patients—including differences in intraocular pressure, tear composition, and inflammation levels—demands highly sensitive and adaptive algorithms to ensure precise, safe, and effective therapeutic outcomes. Standardized feedback thresholds and control strategies remain undeveloped. Chronic implantation or long-term wear also imposes mechanical and biological burdens: micro- and nanoscale devices may cause subtle tissue damage, local fibrosis, or stress on the cornea and retina, while wearable systems can result in discomfort, corneal hypoxia, or altered tear dynamics. Signal accuracy and robustness are critical, as ocular physiological signals are weak and susceptible to interference from light, movement, or tear composition, and sensor drift or failure over time may impair closed-loop control precision. Data security and patient privacy are additional concerns, given real-time wireless transmission and cloud-based processing requirements, all under strict regulatory oversight. Finally, scalability and cost-effectiveness pose practical limitations: highly integrated closed-loop systems are expensive and complex to manufacture, and experience with large-scale clinical trials and standardized certification remains limited. Ultimately, the successful clinical translation of fourth-generation ocular systems will depend on the establishment of standardized chronic safety protocols, adaptive energy management, and robust AI-driven control architectures that collectively ensure long-term safety, comfort, and therapeutic precision.

## 7 Future perspectives of closed-loop controlled ocular biomaterials

### 7.1 AI-augmented therapeutic autonomy

The integration of AI, particularly machine learning and deep neural networks, will be pivotal in enabling predictive, adaptive closed-loop ocular systems. By continuously decoding complex biosignals, AI engines can generate dynamic treatment schedules that self-optimize in response to patient-specific physiological feedback. Future platforms may feature embedded or cloud-linked AI modules that facilitate real-time therapeutic recalibration, moving beyond static thresholds toward true autonomous homeostasis [234]. Beyond conventional AI algorithms, the evolution toward embodied AI could further transform closed-loop ocular biomaterial systems. In this context, “embodiment” refers to endowing therapeutic platforms with physical interfaces and actuation capabilities that directly interact with ocular tissues in real time. Future ophthalmic devices may integrate microscale actuators, flexible piezoelectric elements, or bioinspired robotic structures with advanced biosensing arrays, enabling autonomous sensing–decision–action cycles within the ocular microenvironment. These embodied systems would not only process multimodal biosignals through embedded AI engines but also physically execute therapeutic responses—such as controlled drug release, targeted electrical stimulation, or mechanical reinforcement—without external operator intervention. Such synergy between AI-driven decision-making and embodied actuation could achieve truly adaptive, precision therapies for dynamic ocular pathologies, setting the stage for self-regulating “micro-therapists” that operate seamlessly within the eye [33].

To illustrate how AI operationalizes this loop, we outline several concrete task–actuator pairings spanning dosing, neuromodulation, and edge inference. The following use cases are forward-looking design concepts proposed in this work to illustrate how AI could operationalize closed-loop control in ocular systems; they are not yet demonstrated end-to-end and would require prospective validation.

Concept 1 — AI-guided anti-VEGF scheduling with drug depots (retina). **Sensing.** Periodic OCT and fundus imaging yield quantitative features (e.g., IRF/SRF volumes, central subfield thickness, hyper-reflective foci, choroidal thickness), plus home-monitoring acuity/contrast; **Inference and control.** A sequence model (e.g., transformer or temporal CNN) estimates short-horizon recurrence risk and recommends the next dosing interval. A model-predictive controller (MPC) translates risk into depot release commands (microvalve/open-loop pulse width) and proposes clinic visits when confidence is low. **Actuation.** Subretinal or intravitreal depot (e.g., biodegradable reservoir) with metered micro-release; optional thermal/ultrasound trigger as override. **Safety guardrails.** Uncertainty-aware outputs (conformal prediction) enforce hard bounds on minimum/maximum intervals; human-in-the-loop confirmation; fail-safe escalation to clinician when predicted edema risk or uncertainty exceeds thresholds. **Evaluation plan.** Primary endpoints: time in edema-free state, injection burden, visual acuity non-inferiority; secondary: clinic utilization, adverse events.

Concept 2 — Reinforcement-learning control of IOP with on-eye sensing (glaucoma).

**Sensing.** Smart CL streams IOP, corneal temperature, blink metrics; optional optic-nerve head deformation (OCT-A/biomechanics) nightly. **Inference and control.** A conservative RL agent (offline-trained, safety-layer constrained) learns a titration policy to minimize 24-h IOP variability and peak excursions subject to dose/charge limits. Policy inputs include recent IOP trajectory, circadian phase, adherence estimates. **Actuation.** Micro-dosed prostaglandin/beta-blocker reservoir in the lens skirt or episcleral neuromodulation (low-charge pulsing) targeting aqueous outflow; policy selects when and how much to release/stimulate. **Safety guardrails.** Supervisory controller enforces pharmacologic washout windows, corneal safety (oxygen flux, wear time), and stimulation charge density; automatic policy fallback to guideline dosing if telemetry drops or uncertainty rises. **Evaluation plan.** Endpoints: % time with IOP in target band, peak reduction, adherence-adjusted efficacy; wearable tolerability and corneal physiology as safety readouts.

### 7.2 Convergent functional integration

Next-generation smart ocular biomaterials are expected to converge sensing, actuation, and communication into a unified interface. Such platforms may simultaneously monitor disease-relevant biomarkers, trigger precisely timed interventions, and wirelessly transmit longitudinal data for clinical oversight. This multifunctional consolidation, achievable through multilayer stacking, stretchable electronics, and bioresponsive polymers, reduces device complexity while enhancing therapeutic precision and patient compliance.

### 7.3 Toward personalized, mechanistically informed therapy

As individual variability in ocular disease phenotypes and treatment response becomes increasingly evident, future biomaterials will adopt a personalized design paradigm. Integration of patient-derived omics profiles, AI–guided material selection, and 3D-bioprinting technologies will enable fabrication of constructs tailored to specific anatomical geometries, molecular signatures, and behavioral habits—ushering in a new era of precision ocular therapeutics.

### 7.4 Eye-on-chip platforms for accelerated translation

Physiomimetic in vitro platforms—such as cornea-on-chip, retina-on-chip, and blood-retina barrier models—are emerging as key tools for evaluating dynamic interactions between smart biomaterials and ocular microenvironments. These systems recapitulate native mechanical, fluidic, and biochemical conditions, allowing real-time screening of therapeutic responsiveness, sensor fidelity, and toxicity under controlled conditions. Their deployment is expected to significantly reduce reliance on animal models, streamline regulatory approval, and accelerate clinical translation.

### 7.5 Governance, safety, and ethical horizons

The growing autonomy and data-intensity of closed-loop ocular systems demand proactive regulatory and ethical oversight. Key priorities include ensuring long-term biocompatibility, maintaining algorithmic transparency, securing data privacy, and establishing standards for equitable access. As AI-driven interventions blur the boundary between device and decision-maker, multidisciplinary collaboration across science, medicine, ethics, and policy will be essential to safeguard patient trust and public interest.

## 8 Conclusion

This review advances a generational framework for ophthalmic biomaterials—progressing from first-generation inert scaffolds to second-generation bioactive constructs and third-generation stimuli-responsive systems—and uses it to motivate a fourth generation oriented toward closed-loop control. The value of this schema is not only historical. It clarifies the design primitives (material chemistry, device physics, and control logic), the functional horizons (from support to sensing and actuation), and the clinical maturity of each stage, thereby providing a common language for co-design across materials science, bioelectronics, and ophthalmology.

Fourth-generation smart ocular platforms are defined by the coordinated assembly of sensing, computation, actuation, and communication into a safety-constrained architecture that can adapt to the eye's microenvironment. Today's prototypes demonstrate the pieces—multimodal biosensing on soft, tissue-compatible interfaces; near-eye/edge controllers; and on-demand actuators for drug delivery or neuromodulation—often operating with human/device-in-the-loop supervision. The destination is verifiable, bounded autonomy: systems that adjust therapy within a prescribed safety envelope, expose their state to clinicians, and degrade gracefully under fault.

From a translational standpoint, near-term opportunities should focus on three use cases: smart-contact-lens–based, IOP-triggered dosing for glaucoma; biomarker-gated anterior-segment depots for anti-inflammatory or anti-angiogenic therapy; and adaptive retinal stimulation guided by on-eye electrophysiology. These map onto existing materials capabilities—oxygen-permeable, anti-fouling hydrogels; tunable bioresorbable depots; and low-charge electrodes—and can run under bounded autonomy with clinician override. Lightweight AI controllers can translate sensor streams into patient-specific, safety-constrained dosing and stimulation policies, providing the decision layer for these platforms. In contrast, fully unsupervised implants or designs that cannot satisfy oxygen-flux and thermal or charge budgets are not currently feasible.

Fourth-generation systems should be engineered against non-negotiable limits: corneal oxygen transmissibility, surface temperature rise during telemetry or actuation, electrode charge density and pH excursions, and particulate or degradation profiles. We recommend that any “smart” ocular device report a minimum performance slate: closed-loop latency, dose or charge accuracy, 30- and 90-day drift, fault-handling behavior, and mean time-to-failure under tear or protein fouling. Validation should proceed along a harmonized sequence—bench testing, then eye-on-chip, ex vivo studies, large-animal studies, and early-human evaluation—with reference tasks (for example, IOP band control or edema-free time in macular disease) and model-agnostic scorecards to enable credible comparison across approaches. Progress depends on materials–electronics–control co-design: mesh size and surface chemistry set sensor noise and biofouling; antenna geometry and substrate modulus set telemetry and comfort; controller policy sets safety envelopes. In-situ calibration and drift compensation should be treated as requirements rather than features, with explicit clinician-override pathways.

Translation will require GMP-compatible soft fabrication, sterilization methods that preserve polymer mechanics, and shelf-life testing tied to hydrolysis and oxidation kinetics. Many platforms will be drug–device–software combinations; early regulatory engagement and adoption of software-as-a-medical-device practices will shorten time to clinic. Given continuous telemetry, plans for privacy-preserving learning, cybersecurity, and post-market performance monitoring should be in place from the outset.

Looking forward, fourth-generation, closed-loop ocular biomaterials should (1) deliver sense–decide–act control within explicit oxygen/thermal/charge safety limits; (2) prioritize three deployable use cases (IOP-triggered glaucoma dosing; biomarker-gated anterior-segment depots; ERG-informed adaptive stimulation); (3) report a common metric set (latency, dose/charge accuracy, drift, fault handling, MTTF) across a harmonized bench-to-human pipeline; and (4) pursue materials–electronics–control co-design with in-situ calibration and clinician override, so that adaptability becomes a verifiable property of therapy, not merely a design claim. With shared benchmarks and transparent validation, closed-loop ocular biomaterials can enable patient-specific, constraint-aware therapy that learns and regulates within verified safety limits.

## CRediT authorship contribution statement

**Pengbo Zhang:** Writing – review & editing, Writing – original draft, Visualization, Conceptualization. **Yan Nie:** Writing – original draft, Visualization. **Xiaofang Wang:** Writing – review & editing, Writing – original draft, Visualization. **Xibo Zhang:** Supervision, Conceptualization. **Longqian Liu:** Supervision, Project administration, Funding acquisition, Conceptualization.

## Ethics approval and consent to participate

Not applicable. This article is a review and does not involve any studies with human participants or animals performed by the authors.

## Declaration of competing interest

The authors declare that they have no known competing financial interests or personal relationships that could have appeared to influence the work reported in this paper.
